# CDK6 inactivation counteracts CALR‐mutant‐induced MPN evolution and sensitizes MPN stem cells to interferon‐α treatment

**DOI:** 10.1002/hem3.70363

**Published:** 2026-05-12

**Authors:** Brian Ringhofer, Wolfram Polzer, Michaela Prchal‐Murphy, Eszter Doma, Sebastian Kollmann, Elisabeth Gamper, Jonatan Kendler, Belinda S. Maw, Eva Zebedin‐Brandl, Juan Li, Anthony R. Green, Maria‐Theresa Krauth, Emir Hadzijusufovic, Daniel Ivanov, Peter Valent, Gregor Hoermann, Thorsten Klampfl, Veronika Sexl, Karoline Kollmann

**Affiliations:** ^1^ Department of Biological Sciences and Pathobiology, Institute of Pharmacology and Toxicology University of Veterinary Medicine Vienna Vienna Austria; ^2^ Department of Physiology and Pharmacology Medical University of Vienna Vienna Austria; ^3^ Department of Haematology, Cambridge Stem Cell Institute, Jeffery Cheah Biomedical Center University of Cambridge Cambridge UK; ^4^ Department of Internal Medicine I, Division of Hematology and Hemostaseology Medical University of Vienna Vienna Austria; ^5^ Ludwig Boltzmann Institute for Hematology and Oncology Medical University of Vienna Vienna Austria; ^6^ MLL Munich Leukemia Laboratory Munich Germany; ^7^ University of Innsbruck Innsbruck Austria

## Abstract

Interferon‐α (IFNα) remains a potent therapeutic option for myeloproliferative neoplasms (MPNs) with an activated JAK/STAT signaling axis. However, variable patient responses highlight the need for optimized combination strategies. Recent studies suggest a link between cyclin‐dependent kinase 6 (CDK6) and IFN signaling. Here, we investigated whether CDK6 inhibition might play a role in IFN responsiveness in MPN cells. Using *CALR* ^*del52*^ knockin mice, we observed that genetic ablation of *Cdk6* resulted in a reduction of spleen weight and platelet counts, while concurrently inducing interferon‐associated transcriptional programs and upregulation of interferon‐alpha receptor 1 (IFNAR1) on MPN cells. CDK6‐deficient *CALR* ^*del52*^ hematopoietic stem and progenitor cells (HSPCs) exhibited increased apoptosis and reduced proliferation upon inflammatory challenge compared to wild‐type *CALR* ^*del52*^ cells, positioning CDK6 as a brake on IFN signaling. Pharmacologic inhibition of CDK6 using palbociclib synergized with pegylated IFNα (pegIFNα), resulting in growth inhibition of MPN cells *in vitro* and *in vivo*. In MPN patient samples, lower CDK6 expression was associated with increased IFNAR1 expression and with stronger responses to the palbociclib/pegIFNα combination. Importantly, dose reduction of both palbociclib and pegIFNα maintained efficacy in MPN samples while minimizing cytotoxicity in control hematopoietic cells, revealing a favorable therapeutic window. These findings highlight the potential of combining CDK6 inhibition with pegIFNα to enhance anti‐neoplastic effects in MPNs and support a novel potential approach to improve MPN therapy.

## Graphical abstract

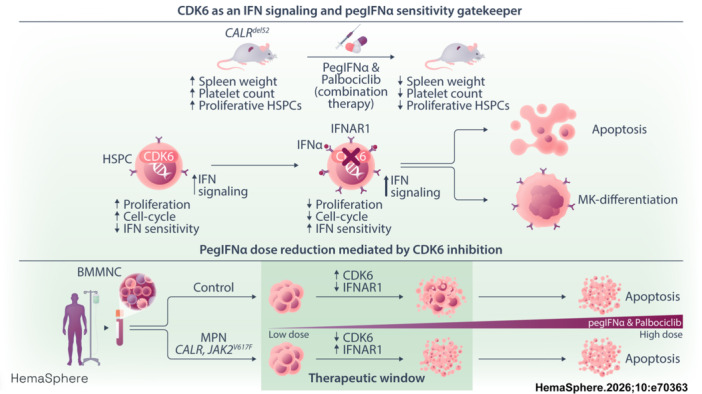

## INTRODUCTION

Myeloproliferative neoplasms (MPNs), including polycythemia vera (PV), essential thrombocythemia (ET), and primary myelofibrosis (PMF), are Philadelphia chromosome‐negative myeloid neoplasms in which neoplastic hematopoietic stem and progenitor cells (HSPCs) produce increased numbers of myeloid cells and/or erythroid cells and/or platelets due to excessive proliferation, clonal expansion, and differentiation.^1^, ^2^, ^3^ Common features of these diseases manifest as bone marrow (BM) hypercellularity, splenomegaly, increased risk of thrombosis, and development of a secondary acute myeloid leukemia (sAML), especially within the PMF subtype.^4^ The latter is associated with poor prognosis and often fatal outcomes.^5^ The underlying cause of MPNs is largely attributed to somatic mutations.^6^, ^7^ While in PV, most patients (above 90%) harbor the *JAK2* ^*V617F*^ gain‐of‐function mutation, only 50% of patients carry this mutation in ET and PMF.^6^, ^8^, ^9^ Within *JAK2*‐non‐mutated patients, about 5%–10% of patients carry a mutation in the thrombopoietin (TPO) receptor gene *MPL*. However, the majority of ET and PMF patients (70%–90%) harbor a mutation in the chaperone protein gene calreticulin (*CALR*), making it the most frequently mutated gene in MPN after *JAK2* ^*V617F*^.^10^, ^11^ *CALR* mutations are characterized by a +1 base pair (bp) frameshift mutation in exon 9 of the gene, frequently a consequence of either a 52 bp deletion (*CALR* ^*del52*^), also known as “Type 1 variant,” or by a 5 bp insertion (*CALR* ^*ins5*^), also called “Type 2 variant.” The +1 frameshift leads to the formation of a novel C‐terminus in the protein and thereby binds to MPL.^7^, ^12^ Involved in various signaling pathways such as JAK‐STAT, the MPL‐CALR complex initiates constitutive signaling leading to excessive proliferation and differentiation of myeloid cells.^7^, ^12^, ^13^, ^14^, ^15^, ^16^

To date, no curative drug therapies for MPN exist. Hydroxyurea, phlebotomy, and aspirin are conventional treatment modalities that moderately improve quality of life.^17^, ^18^ Next to stem cell transplantation, providing promising outcomes but at the expense of significant treatment burden,^19^ novel treatment approaches, such as the use of JAK inhibitors, including ruxolitinib, have shown promising therapeutic potential, especially in MF patients.^20^, ^21^ However, treatment with JAK inhibitors often leads to resistance and toxicity.^17^, ^18^, ^22^ Interferon‐α (IFNα), an inflammatory cytokine, has been a commonly administered therapy in MPN patients for about three decades; however, its broad clinical utility has been limited by high toxicity and frequent dosing schedules. The new (peg) IFNα alternatives^17^, ^22^ were found to have a favorable toxicity profile, increased efficacy, longer stability in the circulation, allowing longer treatment intervals, as well as an overall improvement in safety and tolerability.^22^ Despite significant advantages, pegylated IFNα (pegIFNα) does not exert major effects in all patients, including limited treatment response, especially in mutated *CALR* patients.^23^ To overcome existing limitations and to improve treatment outcomes, pegIFNα‐based combination therapies are of high interest.

Targeting cyclin‐dependent kinase 6 (CDK6) in hematologic diseases has gained increasing attention in recent years. CDK6 is a critical regulator of cell cycle progression and transcriptional regulation,^24^ especially in HSPCs,^25^, ^26^ including neoplastic stem cells.^27^, ^28^ Although CDK6 and its close homolog CDK4 exert similar roles in the cell cycle, CDK6 has several additional kinase‐dependent and independent functions. Among these are mechanisms involved in leukemic transformation^29^, ^30^, ^31^, ^32^ and effects on hematopoietic stem cells (HSCs), such as homing, maintenance, and fitness.^25^, ^33^ Consequently, the clinical efficacy of CDK4/6 inhibitors such as palbociclib, which is approved by the FDA for HR+/HER2− breast cancer treatment,^34^, ^35^ has been extensively studied in the field of hematologic malignancies.^36^ CDK4/6 inhibitors are currently being tested in clinical trials in patients with AML, especially in combination therapies.^37^, ^38^ Recently, CDK6 has been shown as a promising target for MPNs in *JAK2* ^*V617F*^ mouse models of PMF‐ and PV‐like disease, showing reduced disease phenotype and progression via inducing NF‐κB networks associated with inflammation and apoptosis,^39^ highlighting a link between CDK6 and Interferon in MPNs.^39^, ^40^, ^41^

Here, we unravel a novel approach for MPN therapy by inactivating CDK6 and stimulating the IFNα receptor concurrently. We show that *CALR* ^*del52*^ knockin mice harboring a *Cdk6* knockout present a diminished MPN disease phenotype, increased intracellular IFN‐mediated response and IFNAR1 levels, as well as induced apoptosis of *CALR*‐mutant HSPCs *in vitro* and *in vivo*. Pharmacological inhibition of CDK6 revealed similar effects, resulting in an increased susceptibility of MPN cells to treatment with pegIFNα *in vivo* and *in vitro* in MPN patient‐derived BM mononuclear cells (BMMNCs). Notably, we provide a broader therapeutic window when treating patient‐derived BMMNCs with lower combinatorial concentrations of palbociclib and pegIFNα, which demonstrated greater efficacy in *CALR*‐mutated patients compared to controls. This offers a potential novel therapeutic strategy for patients requiring higher effective doses of pegIFNα.

## MATERIALS AND METHODS

### Animals

C57BL/6N (*Cdk6* ^+/+^ *)* and *Cdk6* ^*−/*−^ ^42^ mice were initially crossed with *VavCre CALR*‐mutant knockin mice (here: *CALR* ^*del52*^),^42^ bred and maintained under special pathogen‐free (SPF) conditions at the Institute of Pharmacology and Toxicology, University of Veterinary Medicine, Vienna, Austria. Young, age‐matched (8–12 weeks) male and female mice were used unless indicated otherwise. For transplantation experiments, NSG (NOD.Cg‐Prkdcscid Il2rgtm 1Wjl/SzJ), mice bred and maintained under the same conditions, were used. All procedures were approved by the institutional ethics and animal welfare committee (BMBWF‐68.205/0174‐V/3b/2018, 2024‐0.826.297 and 2022‐0.404.452) and the national authority according to §§26ff. of the Animal Experiment Act, Tierversuchsgesetz 2012 ‐ TVG 2012.

### Primary patient‐derived samples

Information and data of the BM sample donors are shown in Table S2. All patients provided written informed consent. Biobanking (EK1184/2014) and studies on patient samples were approved by the Ethics Committee of the Medical University of Vienna (2415/2024).

### Primary patient‐derived transcriptomic data

RNA‐sequencing data had been published previously and deposited in the Gene Expression Omnibus database (accession number GSE277354).^43^

### Statistics

The appropriate statistical methods were used based on testing for homogeneity of variance and normal distribution. The statistical tests were performed in GraphPad Prism and R‐Studio. For statistical analysis of patient treatments, a Gaussian mixed‐effects model was fitted, using the R package glmmTMB. Log2 fold‐changes were modeled as a function of treatment, genotype, and their interaction as fixed effects and a random intercept for patient (i.e., log2FC ~ genotype × treatment + [1|patient]). Emmeans package was used to estimate marginal means and calculate pairwise treatment comparisons within each genotype. The p‐values were corrected for multiple testing using Tukey's method.

### Ropeginterferon alfa2‐b (pegIFNα)

The active substance Ropeginterferon alfa‐2b (=ropepegIFNα2b=pegIFNα) used in this study was obtained from commercially available pharmaceutical sources. The study was initiated and conducted independently, without involvement of the patent holder or marketing authorization holder. The experiments were solely academic in nature and not intended for product development or commercial use.

### *In vivo* experiments

#### pIpC treatment

*Cdk6* ^+/+^ *CALR* ^+/+^, *Cdk6* ^−/−^ *CALR* ^+/+^, *Cdk6* ^+/+^ *CALR* ^*del52*^, and *Cdk6* ^−/−^ *CALR* ^*del52*^ mice were injected with 10 mg/kg polyinosinic‐polycytidylic acid (pIpC) intraperitoneally (ip) every second day, followed by a 2‐day pause, for a total of 3 weeks. Control mice were injected with the same volumes using phosphate‐buffered saline (PBS). After 3 weeks, mice were dissected, and peripheral blood (PB) parameters were measured using vetABC (veterinary animal blood counter). Spleen, PB, and BM cells were analyzed by flow cytometry.

#### BM transplantation into NSG recipients

In total, 2 × 10^6^ total BM cells from *Cdk6* ^+/+^ *CALR* ^*del52*^ and *Cdk6* ^*−/−*^ *CALR* ^*del52*^ mice (donor) were transplanted into NSG (NOD.Cg‐Prkdcscid Il2rgtm 1Wjl/SzJ) mice (host) via intravenous (iv) injection. After 4 weeks, a blood drop was collected by *vena facialis* puncture, and platelet counts were analyzed using vetABC. Control mice were not injected with BM cells. Nine weeks post‐transplantation, after disease development, mice were dissected, and PB parameters were measured via vetABC. Spleen, PB, and BM cell analyses were performed by flow cytometry.

#### BM transplantation into NSG recipients: Palbociclib/ropegIFNα2b treatment

In total, 2 × 10^6^ total BM cells from *Cdk6* ^+/+^ *CALR* ^*del52*^ were transplanted into NSG (NOD.Cg‐Prkdcscid Il2rgtm 1Wjl/SzJ) mice, as described in the previous paragraph. Nine weeks post‐transplantation and disease development, mice were treated orally (po) once daily with 1 mg/kg palbociclib (PF‐00080665; Pfizer) or PBS. Additionally, mice were injected with 600 ng/mouse pegIFNα (ropegIFNα2b) or PBS subcutaneously (sc) once a week. The treatments were done for a total of 3 weeks, either in combination (palbociclib/pegIFNα) or as single treatments alone.

### Patient samples experiments

#### Liquid culture: Patient samples

In total, 4 × 10^5^ BMMNCs (Tables S2 and S3) from patients with *JAK2* ^*V617F*^ or *CALR‐*mutated MPN and control patients were cultured in Iscove's Modified Dulbecco's Medium (IMDM) supplemented with 5% fetal calf serum (FCS), 15 mM methoxytriglycol (MTG), 2 mM l‐glutamine, 100 ng/mL human stem cell factor (hSCF), 10 ng/mL human interleukin‐3 (hIL‐3), 25 ng/mL human thrombopoietin (hTPO), and 5 U/mL erythropoietin (EPO) for 3 days.^44^ Subsequently, cells were harvested and analyzed by flow cytometry for expression of CD34, CD38, Annexin V/DAPI, intracellular CDK6, IFNAR1, and IFNAR2 (Table S1).

#### Colony formation assay: Patient samples

In total, 5 × 10^4^ BMMNCs from *JAK2* ^*V617F*^, *CALR* Type 1, *CALR* Type 2, and control patients (listed in Tables S2 and S3) were cultured in fully supplemented human methylcellulose (MethoCult H4435 Enriched). Cells were resuspended in 2 mL of this medium in 35‐mm dishes and incubated at 37°C, 5% CO_2_ for 7–14 days.^44^ Colonies were counted by using an OLYMPUS IX71 inverted microscope, harvested, and analyzed via flow cytometry using CD34, CD38, CD41, and CD42 surface antibodies (Table S1).

## RESULTS

### *Cdk6* knockout diminishes MPN disease phenotype in *CALR* ^*del52*^ knockin mice

**Figure 1:**
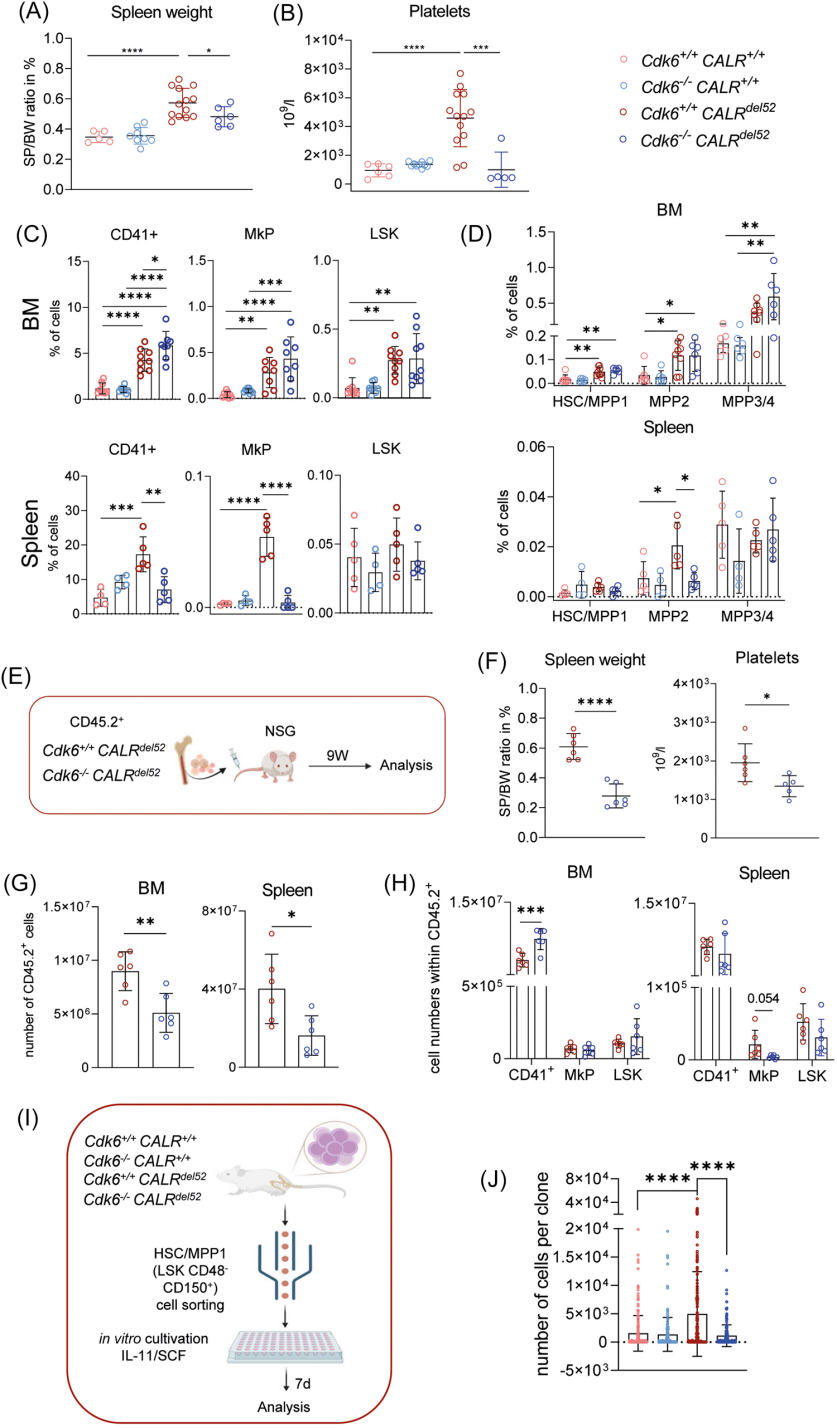
***Cdk6* loss reduces the myeloproliferative neoplasm (MPN) phenotype in *CALR*‐mutant mice in a cell‐intrinsic manner. (A)** Spleen (SP) weight to body weight (BW) ratio in % and **(B)** thrombocyte (platelet) counts in 10^9^/L of 8‐week‐old *Cdk6* ^+/+^ *CALR* ^+/+^ (light red, *n* = 6), *Cdk6* ^−/−^ *CALR* ^+/+^ (light blue, *n* = 8), *Cdk6* ^+/+^ *CALR* ^*del52*^ (red, *n* = 13), and *Cdk6* ^−/−^ *CALR* ^*del52*^ (blue, *n* = 6) mice. **(C)** Flow cytometric analysis of bone marrow (BM, upper panel) and SP (lower panel) in 8‐week‐old *Cdk6* ^+/+^ *CALR* ^+/+^, *Cdk6* ^−/−^ *CALR* ^+/+^, *Cdk6* ^+/+^ *CALR* ^*del52*^, and *Cdk6* ^−/−^ *CALR* ^*del52*^ showing MKs (megakaryocytes, CD41^+^), MkP (megakaryocyte progenitor, Lin^−^ Sca‐1^+^ c‐Kit^+^ CD41^+^ CD150^+^), and LSK (Lin^−^ Sca‐1^+^ c‐Kit^+^) % of total populations. BM *n* ≥ 8, SP *n* ≥ 2. **(D)** Flow cytometric analysis of BM (upper panel) and SP (lower panel) in 8‐week‐old *Cdk6* ^+/+^ *CALR* ^+/+^, *Cdk6* ^−/−^ *CALR* ^+/+^, *Cdk6* ^+/+^ *CALR* ^*del52*^, and *Cdk6* ^−/−^ *CALR* ^*del52*^ showing hematopoietic stem cell (HSC)/MPP1 (Lin^−^ Sca‐1^+^ c‐Kit^+^ CD150^+^ CD48^−^), MPP2 (Lin^−^ Sca‐1^+^ c‐Kit^+^ CD150^+^ CD48^+^), and MPP3/4 (Lin^−^ Sca‐1^+^ c‐Kit^+^ CD150^−^ CD48^+^) % of total populations. BM *n* ≥ 4, SP *n* ≥ 2. Error bars represent mean±SD. *P < 0.05; **P < 0.01; ***P < 0.001; and ****P < 0.0001 by ordinary one‐way analysis of variance (ANOVA) followed by Tukey's multiple comparison test. **(E)** Experimental scheme: transplantation of 2 × 10^6^ BM cells of 8‐week‐old *Cdk6* ^+/+^ *CALR* ^*del52*^ (red) and *Cdk6* ^−/−^ *CALR* ^*del52*^ (blue) mice (CD45.2^+^) into NSG recipient mice (CD45.1^+^) via intravenous (iv) injection. Mice were analyzed 9 weeks post‐injection, when the disease had developed. Created in BioRender. Kollmann, K. (2026) https://BioRender.com/m4ljak5 **(F)** SP weight to BW ratio in % (left) and thrombocyte (platelet) counts in 10^9^/L (right) of NSG recipient mice 9 weeks post‐injection received *Cdk6* ^+/+^ *CALR* ^*del52*^ (red, *n* = 6) and *Cdk6* ^−/−^ *CALR* ^*del52*^ (blue, *n* = 6) donor BM. **(G)** Donor chimerism. Flow cytometric analysis of NSG recipient mice with *Cdk6* ^+/+^ *CALR* ^*del52*^ (*n* = 6) and *Cdk6* ^−/−^ *CALR* ^*del52*^ (*n* = 6) donor cells showing total cell numbers of engrafted cells (CD45.2^+^) in the BM (left) and SP (right). **(H)** Flow cytometric analysis of LSK, MkP, and MK populations within NSG recipient mice with *Cdk6* ^+/+^ *CALR* ^*del52*^ (red, *n* = 6) and *Cdk6* ^−/−^ *CALR* ^*del52*^ (blue, *n* = 6) donor cells showing total cell numbers of CD41^+^, MkP, and LSK within engrafted cells (CD45.2^+^) in the BM (left) and SP (right). Error bars represent mean±SD. *P < 0.05; **P < 0.01; ***P < 0.001; and ****P < 0.0001 by unpaired two‐tailed Students *t*‐test. **(I)** Experimental scheme: single‐cell sorting of HSC/MPP1 cells from BM of 8‐week‐old *Cdk6* ^+/+^ *CALR* ^+/+^ (light red), *Cdk6* ^−/−^ *CALR* ^+/+^ (light blue), *Cdk6* ^+/+^ *CALR* ^*del52*^ (red), and *Cdk6* ^−/−^ *CALR* ^*del52*^ (blue) mice (*n* = 2 per group) in StemSPAN SFEM II HSC expansion media with human interleukin‐11 (hIL‐11) and stem cell factor (SCF). Clones were analyzed 7 days post‐sorting. Created in BioRender. Kollmann, K. (2026) https://BioRender.com/blyzfnc **(J)** Total cell numbers (per clone) measured via flow cytometry after 7 days of culture; 192 wells (each well representing a clone) per genotype (2 mice each) were analyzed, and total cell numbers derived from each clone were plotted (number of cells per clone). Error bars represent mean±SD. ****P < 0.0001 by ordinary one‐way ANOVA followed by Tukey's multiple comparison test.

To study the effects of *Cdk6* on MPNs harboring a Type 1 *CALR* mutation, we crossed *Cdk6* ^−/−^ (C57BL/6) mice^45^ with *CALR* ^*del52*^ *VavCre* knockin mice.^42^ In line with previously published data, adult *Cdk6* ^+/+^ *CALR* ^*del52*^ mice displayed thrombocytosis, splenomegaly, and BM hypercellularity when compared to wild‐type (*Cdk6* ^+/+^ *CALR* ^+/+^) mice. *CALR*‐mutant mice devoid of *Cdk6* (*Cdk6* ^−/−^ *CALR* ^*del52*^) exhibited a smaller spleen size and lower platelet counts between 8 and 10 weeks of age compared to *Cdk6* ^+/+^ *CALR* ^*del52*^ mice (Figure 1A,B), vividly underscoring a significantly diminished MPN phenotype. No signs of fibrosis have been observed at this disease stage. No changes were observed in other blood parameters, except for red blood cell (RBC) counts (Figure S1A). Flow cytometric analysis presented a significant increase of CD41^+^ megakaryocyte (MK) populations in the BM but a reduction of the MKs and MK progenitor (MkP) cells in the spleens of *CALR*‐mutant mice lacking *Cdk6* compared to *Cdk6* ^+/+^ CALR^del52^ mice (Figure 1C). This finding was validated by hematoxylin & eosin (H&E) stainings (Figure S1B,C). Erythroid, myeloid, or lymphoid populations did not show major CDK6‐dependent alterations in BM, spleen, or PB of *CALR*‐mutant mice (Figure S1D).

Although relative numbers of Lineage^−^ c‐Kit^+^ Sca‐1^+^ (LSK) cells did not differ in the absence of *Cdk6* (Figure 1C), the composition of HSPC subsets was altered. Whereas the MPP3/4 subpopulation was slightly increased in the BM of *Cdk6* knockout mice, MPP2 cells were significantly less present in the spleens of *Cdk6* ^−/−^ *CALR* ^*del52*^ versus *Cdk6* ^+/+^ *CALR* ^*del52*^ mice (Figure 1D). MPP2 cells have been shown to preferentially differentiate into the MK‐lineage and ultimately lead to platelet production,^46^, ^47^ while MPP3/4 populations can differentiate into myeloid precursors and inflammatory cells,^46^, ^48^ suggesting that the consequences of *Cdk6* loss might influence early stages of MK‐differentiation.

To evaluate whether the effect of *Cdk6* deletion in *CALR‐*mutant mice is cell intrinsic, we transplanted total BM cells from *Cdk6* ^+/+^ *CALR* ^*del52*^ and *Cdk6* ^−/−^ *CALR* ^*del52*^ mice into NSG recipients (Figure 1E). Nine weeks post‐injection, the disease was fully developed, and the mice were analyzed. Comparable to *Cdk6* ^−/−^ *CALR* ^*del52*^ mice, spleen size and platelet counts were significantly reduced in recipients of *Cdk6* ^−/−^ *CALR* ^*del52*^ BM compared to recipients of *Cdk6* ^+/+^ *CALR* ^*del52*^ BM (Figure 1F). Independent of the *CALR*‐mutant background, engraftment of transgenic BM into NSG recipients was reduced in the absence of *Cdk6* (Figure 1G), consistent with previous findings.^33^ In line with observations in donor mice, BM from *Cdk6* ^−/−^ *CALR* ^*del52*^ recipients revealed a significant increase of MKs in the BM compared to *Cdk6* ^+/+−^ *CALR* ^*del52*^ but a decrease of MkPs in the spleen, while LSK populations appeared unaffected by CDK6 (Figure 1H). Despite reduced donor cell chimerism in *Cdk6* ^−/−^ *CALR* ^*del52*^ recipients, the comparable disease phenotype suggests that changes in MK and HSPC populations are not solely due to altered homing but may instead reflect cell‐intrinsic effects.

To explore this further, we assessed the effect of CDK6 on proliferation and differentiation in *CALR*‐mutant HSPCs by culturing single HSC/MPP1 cells from *Cdk6* ^+/+^ *CALR* ^+/+^, *Cdk6* ^−/−^ *CALR* ^+/+^, *Cdk6* ^+/+^ *CALR* ^*del52*^, and *Cdk6* ^−/−^ *CALR* ^*del52*^ mice in the presence of SCF and rmIL‐11,^49^ supporting self‐renewal of HSCs and early lineage‐biased differentiation (Figure 1I). After 7 days, *CALR*‐mutant cells without CDK6 displayed significantly fewer cells per clone compared to their counterparts with CDK6, similar to wild‐type *Cdk6* ^+/+^ *CALR* ^+/+^ cells (Figures 1J and S1E). *Cdk6* ^−/−^ *CALR* ^*del52*^ cells were not able to form large colonies above 10.000 cells, while up to 20% of *Cdk6* ^+/+^ *CALR* ^*del52*^ cells did form such larger colonies (Figures 1J and S1F). Although colony size was significantly altered in cells lacking *Cdk6*, flow cytometric analysis revealed that the loss of CDK6 had no major effect on the LSK population, whereas cells harboring the *CALR* mutation displayed reduced LSK percentages (Figure S1G). While *Cdk6* ^−/−^ cells without the *CALR* mutation differentiated into more lineage‐positive (myeloid/lymphoid) cells, as previously published,^25^, ^39^ but less into the MK‐lineage, these effects were not visible in cells harboring the *CALR* ^*del52*^ mutation (Figure S1H,I). Taken together, these results suggest that *Cdk6* loss significantly reduces self‐renewal and proliferation in neoplastic HSPCs but does not alter differentiation in a mutant *CALR* background *in vitro*.

### *Cdk6* loss upregulates interferon‐associated transcriptional programs in MkPs

**Figure 2:**
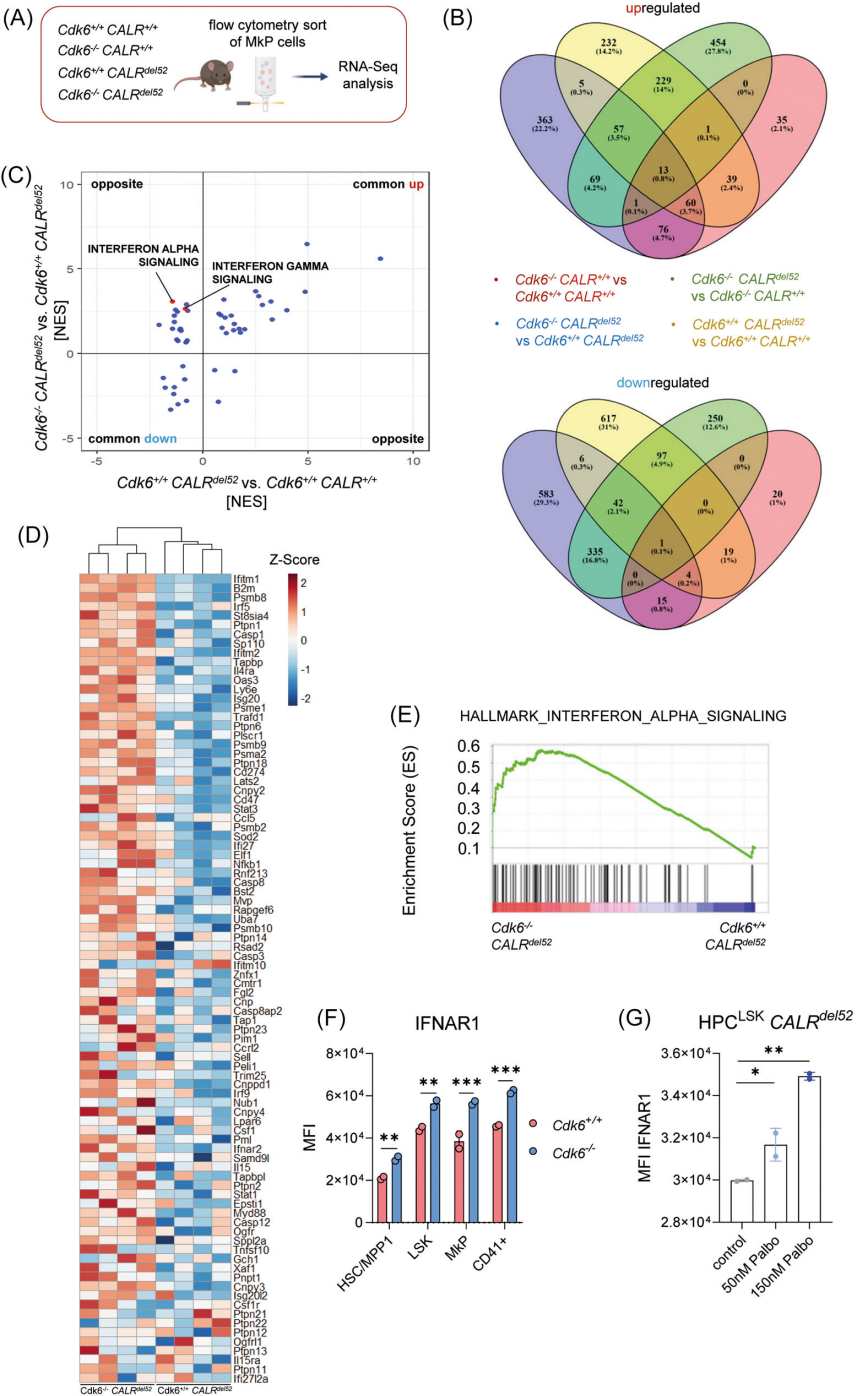
***Cdk6* knockout induces transcriptomic interferon (IFN) signaling pathways in *CALR*‐mutant megakaryocyte progenitors (MkPs). (A)** Experimental scheme: MkP (Lin^−^ Sca‐1^+^ c‐Kit^+^ CD41^+^ CD150^+^) cells from *Cdk6* ^+/+^ *CALR* ^+/+^, *Cdk6* ^−/−^ *CALR* ^+/+^, *Cdk6* ^+/+^ *CALR* ^*del52*^, and *Cdk6* ^−/−^ *CALR* ^*del52*^ mice (*n* = 4 per group) were sorted using fluorescence‐activated cell sorting (FACS), followed by RNA‐isolation and RNA‐Seq. Created in BioRender. Kollmann, K. (2026) https://BioRender.com/g43ll2y **(B)** Venn diagram showing overlaps of common or distinct deregulated genes obtained from RNA‐Seq between *Cdk6* ^+/+^ *CALR* ^+/+^, *Cdk6* ^−/−^ *CALR* ^+/+^, *Cdk6* ^+/+^ *CALR* ^*del52*^, and *Cdk6* ^−/−^ *CALR* ^*del52*^ gene sets, giving a total of 3623 deregulated genes. Blue—significantly up‐ or downregulated genes in *Cdk6* ^−/−^ *CALR* ^*del52*^ versus *Cdk6* ^+/+^ *CALR* ^*del52*^ (*Cdk6* knockout effect in mutant background), orange—significantly up‐ or downregulated genes in *Cdk6* ^+/+^ *CALR* ^*del52*^ versus *Cdk6* ^−/−^ *CALR* ^+/+^ (mutant effect), green—significantly up‐ or downregulated genes in *Cdk6* ^−/−^ *CALR* ^*del52*^ versus *Cdk6* ^−/−^ *CALR* ^+/+^ (mutant effect in *Cdk6* knockout background), red—significantly up‐ or downregulated genes in *Cdk6* ^+/+^ *CALR* ^+/+^ versus *Cdk6* ^−/−^ *CALR* ^+/+^ (*Cdk6* knockout effect). Deregulated genes were chosen by a false discovery rate (FDR) < 0.1 threshold. **(C)** Scatterplot of *Cdk6* ^−/−^ *CALR* ^*del52*^ versus *Cdk6* ^+/+^ *CALR* ^*del52*^ (*y*‐axis) and *Cdk6* ^+/+^ *CALR* ^*del52*^ versus *Cdk6* ^+/+^ *CALR* ^+/+^ (*x*‐axis) datasets showing common and distinct (opposite) regulated pathway enrichment. Interferon‐alpha and interferon‐gamma signaling pathways are highlighted as top distinct pathways, upregulated in *Cdk6* ^−/−^ *CALR* ^*del52*^ versus *Cdk6* ^+/+^ *CALR* ^*del52*^ and downregulated in *Cdk6* ^+/+^ *CALR* ^*del52*^ versus *Cdk6* ^+/+^ *CALR* ^+/+^. **(D)** Heatmap displaying deregulated genes significantly enriched within the interferon‐alpha and interferon‐gamma signaling pathways in the *Cdk6* ^−/−^ *CALR* ^*del52*^ versus *Cdk6* ^+/+^ *CALR* ^*del52*^ dataset. Plotted are variance‐stabilized and batch‐corrected expression values, scaled by row. **(E)** Enrichment plot from gene set enrichment analysis (GSEA) using HALLMARK‐database, revealing interferon‐alpha signaling as a top upregulated pathway in the *Cdk6* ^−/−^ *CALR* ^*del52*^ versus *Cdk6* ^+/+^ *CALR* ^*del52*^ dataset. **(F)** Flow cytometric analysis of interferon‐alpha receptor 1 (IFNAR1) in *Cdk6* ^+/+^ (red) and *Cdk6* ^−/−^ (blue) hematopoietic stem cell (HSC)/MPP1, MkP, and CD41^+^ cell populations (*n* = 2 per group). Error bars represent mean ± SD. **P < 0.01; ***P < 0.001 by unpaired two‐tailed Student's *t*‐test. **(G)** IFNAR1 expression on murine HPC^LSK^ *CALR* ^*del52*^ cells upon palbociclib treatment. Concentrations of 50 and 150 nM palbociclib (PD‐0332991) or phosphate‐buffered saline (PBS) control were used. *n* = 2 per group. Error bars represent mean ± SD. *P < 0.05; **P < 0.01 by ordinary one‐way analysis of variance (ANOVA) followed by Tukey's multiple comparison test.

To identify the molecular consequences resulting from the loss of *Cdk6* in *CALR*‐mutant MkPs, resembling the most affected lineage in this disease, we performed RNA‐sequencing in MkPs from *Cdk6* ^+/+^ *CALR* ^+/+^, *Cdk6* ^−/−^ *CALR* ^+/+^, *Cdk6* ^+/+^ *CALR* ^*del52*^, and *Cdk6* ^−/−^ *CALR* ^*del52*^ mice (Figure 2A). Differential gene expression analysis revealed 3623 deregulated genes between all genotypes when setting an adjusted P‐value of P < 0.05. *Cdk6* loss in the *CALR*‐mutant background showed 647 up‐ and 986 downregulated genes compared to 225 up‐ and 59 downregulated genes in the wild‐type setting (Figure 2B). In total, 170 of the 284 genes significantly deregulated between *Cdk6* ^−/−^ *CALR* ^+/+^ and *Cdk6* ^+/+^ *CALR* ^+/+^ MkPs (red) were seen similarly deregulated in *Cdk6* ^−/−^ *CALR* ^*del52*^ versus *Cdk6* ^+/+^ *CALR* ^*del52*^ (blue) comparisons. Gene set enrichment analysis (GSEA) revealed that the most enriched pathways were commonly upregulated in the *Cdk6* ^−/−^ *CALR* ^*del52*^ versus *Cdk6* ^+/+^ *CALR* ^*del52*^ (*y*‐axis, *Cdk6* effect) and *Cdk6* ^+/+^ *CALR* ^*del52*^ versus *Cdk6* ^+/+^ *CALR* ^+/+^ (*x*‐axis, *CALR* ^*del52*^ effect) datasets, including the p53 pathway (Figure S2A), which is in line with previous studies.^31^, ^50^, ^51^ Strikingly, IFNα and IFNγ‐related pathways were among the most significantly upregulated pathways in *Cdk6* ^−/−^ MkPs harboring the *CALR* mutation compared to their controls (Figures 2C–E and S2A). Downregulation of these pathways in *CALR*‐mutant MkPs compared to wild‐type, highlights that the enrichment of IFN‐associated gene sets seems to be a counteractive mechanism caused by *Cdk6* loss (Figures 2C–E and S2B). In order to better understand differences of CDK6 loss on MkPs of the BM versus the spleen, we performed RNA‐sequencing in MkPs from *Cdk6* ^+/+^ *CALR* ^*del52*^ and *Cdk6* ^−/−^ *CALR* ^*del52*^ spleens. Overall, *Cdk6* loss seems to have different effects on MkPs depending on the tissue. Whereas the upregulation of the IFN‐associated pathways was pronounced in the BM, spleen‐derived MkPs revealed downregulated E2F and G2M checkpoint pathways (Figure S2C). Among the genes which had the largest influence on the IFN pathway enrichment in BM MkPs were not only classical IFN genes such as IFNAR, STAT1, or ISGs, but also caspases, which play a role in apoptosis (Figure 2D,E), aligning with studies of IFN‐induced apoptotic cell death.^52^, ^53^ We confirmed the upregulation of the IFN‐associated mediator interferon‐alpha receptor 1 (IFNAR1), a critical player in IFN‐mediated signaling, in *Cdk6* ^−/−^ cells by flow cytometry. An increased level of IFNAR1 expression was observed in HSPC and MK‐associated subpopulations of *Cdk6* knockout mice compared to wild‐type controls (Figure 2F). In HPC^LSK^ cell lines overexpressing *CALR* ^*del52*^, treatment with the clinically approved CDK4/6 inhibitor palbociclib (PD‐0332 991) resulted in increased expression of IFNAR1 (Figure 2G). These findings highlight that CDK6 deletion or inhibition may render MkP cells more IFN‐responsive.

In line, previous studies described MPN HSPC subsets with higher IFN response by CD41 and MHCII expression. In these studies, the expression of the MK marker CD41 as well as MHCII was negatively correlated with cell cycle inhibition, apoptosis, and higher response to IFN.^54^, ^55^ We were therefore wondering if our transcriptomic data from *Cdk6*‐ablated *CALR* ^*del52*^ MkPs display similar observations. Using the published dataset,^55^ we identified sets of genes highly expressed in CD41‐low (CD41^lo^) expressing cells. These CD41^lo^ signatures were enriched in *Cdk6* ^−/−^ *CALR*‐mutant MkP cells, highlighting reduced cell cycle activity and increased susceptibility to IFN stimulation (Figure S2D). Similarly, MHCII^lo^ and MHCII^hi^ HSC subpopulations in *CALR*‐mutant MPNs revealed that STAT1‐mediated IFN signaling and therefore MHCII expression in HSPC subsets are associated with reduced cycling, increased differentiation, and apoptosis.^54^ *Cdk6* ^−/−^ *CALR* ^*del52*^ MkPs displayed significant upregulation of those MHCII‐associated signature genes when compared to *Cdk6* ^+/+^ *CALR* ^*del52*^ MkPs (Figure S2E).

Together, these data indicate that CDK6 plays a role in IFN signaling in the context of *CALR* mutations, and that loss of CDK6 pushes cells into an IFN‐responsive transcriptional state resembling CD41^lo^ or MHCII^hi^ HSPC populations in MPN patients.

### *Cdk6* deficiency in conjunction with pIpC treatment enhances cell death in *CALR‐*mutated HSPCs

**Figure 3:**
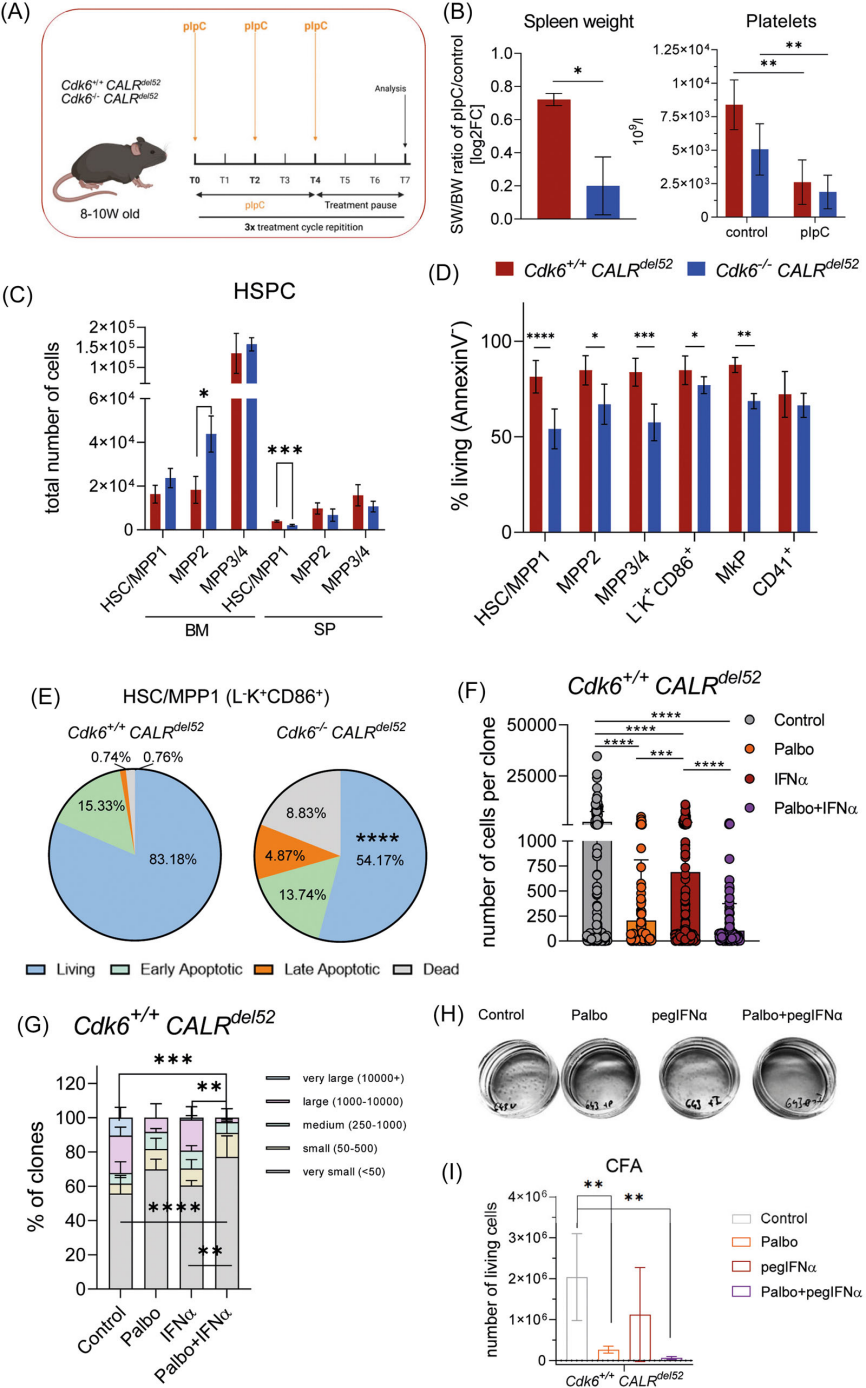
**CDK6 inactivation increases interferon‐induced apoptosis and reduces proliferation in *CALR* ^*del52*^ hematopoietic stem and progenitor cells (HSPCs). (A)** Experimental scheme: 8–10‐week‐old *Cdk6* ^+/+^ *CALR* ^*del52*^ and *Cdk6* ^−/−^ *CALR* ^*del52*^ mice were treated with polyinosinic‐polycytidylic acid (pIpC) via intraperitoneal (ip) injection every 48 h, followed by a 3‐day pause for a total of 3 weeks. After 21 days, mice were sacrificed, blood parameters were measured, and bone marrow (BM) and spleen (SP) were analyzed. Created in BioRender. Kollmann, K. (2026) https://BioRender.com/j70j3f2 **(B)** Log2 fold‐change of SP weight to BW ratio in % over phosphate‐buffered saline (PBS)–injected controls (left) and thrombocyte (platelet) counts in 10^9^/L (right) of 8–10‐week‐old *Cdk6* ^+/+^ *CALR* ^*del52*^ and *Cdk6* ^−/−^ *CALR* ^*del52*^ mice injected with PBS (control) or pIpC. **(C)** Flow cytometric analysis showing total cell numbers of HSPC subsets within BM and SP of pIpC‐treated *Cdk6* ^+/+^ *CALR* ^*del52*^ and *Cdk6* ^−/−^ *CALR* ^*del52*^ mice. HSPC (from L^−^K^+^CD86^+^) populations comprise HSC/MPP1, MPP2, and MPP3/4 cells. **(D)** Annexin‐V/DAPI flow cytometry staining showing percentages of living (Annexin‐V^−^ DAPI^−^) HSC/MPP1, MPP2, MPP3/4, L^−^K^+^CD86^+^, MkP, and CD41^+^ cells in the BM of pIpC‐treated *Cdk6* ^+/+^ *CALR* ^*del52*^ and *Cdk6* ^−/−^ *CALR* ^*del52*^ mice. Error bars represent mean ± SD. *n* ≥ 2. *P < 0.05; **P < 0.01; ***P < 0.001; and ****P < 0.0001 by unpaired two‐tailed Student's *t*‐test. **(E)** Representative pie‐chart of Annexin‐V/DAPI apoptosis staining on pIpC‐treated HSC/MPP1 (Lin^−^ c‐Kit^+^ CD86^+^ CD150^+^ CD48^−^) in *Cdk6* ^+/+^ *CALR* ^*del52*^ and *Cdk6* ^−/−^ *CALR* ^*del52*^ mice. **(F)** Flow cytometric analysis for proliferation of HSC/MPP1 single‐cell–sorted clones from *Cdk6* ^+/+^ *CALR* ^*del52*^ mouse BM after 7 days of culturing in StemSPAN SFEM II HSC expansion media with human interleukin‐11 (hIL‐11) and stem cell factor (SCF), treated with PBS control (gray), 200 nM palbociclib (orange), 100 U interferon‐α (IFNα) (red), or a 200 nM palbociclib/100 U IFNα combination (purple); 48 wells (each well representing a clone) per treatment condition were analyzed (192 total). For each treatment (*n* = 2), all cell numbers derived from each clone were plotted (number of cells per clone). Error bars represent mean ± SD. ***P < 0.001; ****P < 0.0001 by RM one‐way analysis of variance (ANOVA) with Geisser‐Greenhouse correction followed by Tukey's multiple comparison test. **(G)** Colony size growth using cell numbers obtained by flow cytometry of HSC/MPP1 single‐cell–sorted clones from *Cdk6* ^+/+^ *CALR* ^*del52*^ mouse BM after 7 days of culturing in StemSPAN SFEM II HSC expansion media with hIL‐11 and SCF, treated with PBS control (gray), 200 nM palbociclib (orange), 100 U IFNα (red), or a 200 nM palbociclib/100 U IFNα combination (purple). For each treatment (*n* = 2), clone sizes were categorized into very small (<50), small (50–250), medium (250–1000), large (1000–10,000), and very large (>10,000) cell numbers, and percentages were calculated. Data are presented with mean ± SD of two independent experiments and *n* = 2 mice per experiment (4 total). **P < 0.01; ***P < 0.001; and ****P < 0.0001 by ordinary two‐way ANOVA followed by Tukey's multiple comparison test. **(H)** Representative picture of colony formation assay (CFA) using 100 bulk‐sorted Lin^−^ c‐Kit^+^ Sca‐1^+^ (LSK) cells from *Cdk6* ^+/+^ *CALR* ^*del52*^ mouse BM treated with PBS control, 200 nM palbociclib, 600 ng pegylated IFNα (pegIFNα), and a 200 nM palbociclib/600 ng pegIFNα combination. **(I)** CFA using fully supplemented murine methylcellulose with 100 bulk‐sorted LSK cells from *Cdk6* ^+/+^ *CALR* ^*del52*^ mouse BM treated with PBS control (gray), 200 nM palbociclib (orange), 600 ng pegIFNα (red), or a 200 nM palbociclib/600 ng pegIFNα combination (purple). Flow cytometric analysis was carried out 7 days post‐cultivation and analyzed for total cell number. Error bars represent mean ± SD. *n* = 3 per condition. *P < 0.05; **P < 0.01 by ordinary one‐way ANOVA followed by Tukey's multiple comparison test.

Next, we sought to examine whether the upregulation of IFN mediators and IFNAR1 renders *Cdk6* ^−/−^ *CALR* ^*del52*^ mice more responsive to IFN stimuli by using pIpC. *Cdk6* ^+/+^ *CALR* ^*del52*^ and *Cdk6* ^−/−^ *CALR* ^*del52*^ mice were treated with pIpC or PBS every 2 days for a total of 3 weeks (Figure 3A). Recurrent pIpC injection led to a significant reduction of splenomegaly and thrombocyte counts, most prominent when *Cdk6* was absent. Notably, these effects were demonstrated as the percent change of spleen weight (SW) to body weight (BW) ratio normalized to control groups (Figure 3B). No signs of fibrosis have been observed in the different genotypes. To prevent unintended interferon‐related effects on the HSPC surface marker Sca‐1, we selected to use CD86 as an alternative to determine LSK populations.^56^ *Cdk6* ^−/−^ *CALR* ^*del52*^ mice showed increased numbers of MK‐biased MPP2 cells in the BM but significantly reduced HSC/MPP1 cells in the spleen when treated with pIpC (Figures 3C and S3A). While no significant differences were observed in L^−^K^+^CD86^+^ HSPCs and MkPs, mice lacking *Cdk6* maintained lower CD41^+^ cell numbers in the spleen but not in the BM (Figure S3B–D). In *Cdk6* knockout mice treated with pIpC, MPP1‐4, L‐K^+^CD86^+^, and MkP cell populations showed a reduction in the number of living cells compared to the controls (Figures 3D and S3E). HSC/MPP1 cells were most affected by apoptosis in pIpC‐treated *CALR*‐mutant mice harboring a *Cdk6* knockout (Figures 3D,E and S3E). Remarkably, the pIpC‐induced apoptosis in HSC/MPP1 cells was observed only in *Cdk6* knockout mice carrying a *CALR* mutation, whereas *Cdk6* knockout mice with wild‐type *CALR* did not exhibit increased cell death upon treatment (Figure S3E). Resembling the homeostatic condition of the *Cdk6* ^−/−^ *CALR* ^*del52*^ MkPs, pIpC‐treated HSC/MPP1 and HSPC cells without *Cdk6* revealed increased IFNAR1 levels, presenting a possible explanation for the pronounced IFN‐induced cell death (Figure S3F). These findings highlight that *CALR* ^*del52*^‐driven cells lacking CDK6 are more prone to inflammation‐induced apoptosis compared to *CALR*‐mutant only, suggesting a potential therapeutic window, where disease‐driving cells are more vulnerable to a treatment than normal cells.

### Pharmacologic CDK6 kinase inhibition enhances IFNα responsiveness

To examine whether these findings can be translated into a more clinical setting, we applied the CDK4/6 kinase inhibitor palbociclib (PD‐0332991) in combination with IFNα to *CALR* ^*del52*^ HSPCs. As expected, treatment of *Cdk6* ^+/+^ *CALR* ^*del52*^ HSC/MPP1 cells with only palbociclib or IFNα alone significantly reduced proliferation and colony size. The strongest effect was observed when both substances were administered in combination (Figure 3F,G). Induced MK‐differentiation and increased IFNAR1 expression were observed in IFNα‐ and especially in combination‐treated HSC/MPP1 single cell colonies (Figure S3G), aligning with previous studies of IFN‐induced megakaryopoiesis and transcriptional upregulation of *IFNAR1*.^57^, ^58^, ^59^, ^60^

The beneficial effect of the combinatorial treatment with palbociclib and IFNα was confirmed in a colony formation assay (CFA) of bulk‐sorted *Cdk6* ^+/+^ *CALR* ^*del52*^ LSK cells using pegIFNα, currently used as a treatment for patients suffering from PV.^22^ Although methylcellulose plating of LSK cells primarily promotes myeloid differentiation rather than MK‐differentiation, palbociclib and palbociclib/pegIFNα combination treatments gave results comparable to the ones observed in HSPCs (Figure 3H,I). Palbociclib/pegIFNα treated LSKs primarily differentiated into the myeloid lineage and hardly affected L^−^K^+^CD86^+^ or L^−^K^+^CD86^−^ populations (Figure S3H). We then assessed the effects of combinatory treatments on MK‐differentiation in the presence of TPO (Figure S3I–K). Similarly, we determined reduced viability and increased CD41^+^ cells in palbociclib/pegIFNα combination‐treated samples, underscoring enhanced cell death and MK‐differentiation when treated with palbociclib and pegIFNα concomitantly (Figure S3J,K). Together, these data suggest that the effect of reduced proliferation, colony size, increased apoptosis, as well as increased IFNAR1 levels in *CALR*‐mutant cells does not rely on cell environment or differentiation lineage, but rather on the presence of CDK6 in immature HSPCs, ultimately allowing stronger IFNα responsiveness of cells when CDK6 is inhibited.

### Combinatorial treatment with CDK4/6 kinase inhibitor and pegIFNα reduces MPN phenotype *in vivo*

**Figure 4:**
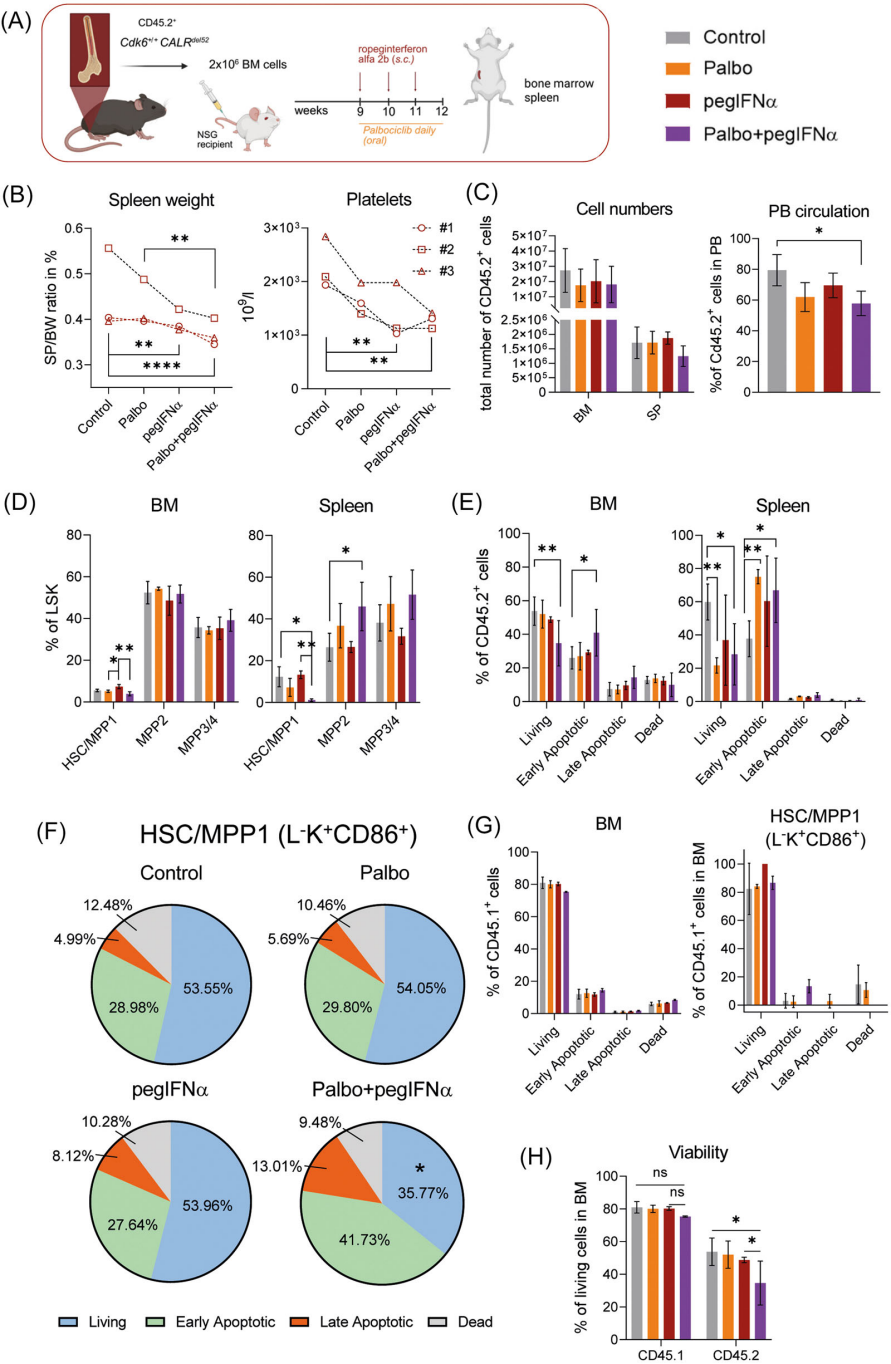
**Myeloproliferative neoplasm (MPN) disease phenotype is reduced using a CDK4/6 inhibition and pegylated interferon‐α (pegIFNα) combinatorial treatment *in vivo.* (A)** Experimental scheme: transplantation of 2 × 10^6^ bone marrow (BM) cells of 8‐week‐old *Cdk6* ^+/+^ *CALR* ^*del52*^ mice (CD45.2^+^) into NSG recipients (CD45.1^+^) via intravenous (iv) injection. After disease development 9 weeks post‐injection, mice were treated daily by oral administration of 50 mg/kg palbociclib and weekly by subcutaneous injection of 600 ng pegIFNα per mouse in combination, or with a phosphate‐buffered saline (PBS) control (oral/subcutaneous injection). After 3 weeks of treatment, peripheral blood (PB), BM, and spleen (SP) of the mice were analyzed by flow cytometry. Created in BioRender. Kollmann, K. (2026) https://BioRender.com/t9q05dk **(B)** SP weight to body weight (BW) ratio in % (left) and thrombocyte (platelet) counts in 10^9^/L (right) of NSG recipient mice transplanted with *Cdk6* ^+/+^ *CALR* ^*del52*^ mouse BM and treated with 50 mg/kg palbociclib (orange), 600 ng pegIFNα (red), combination (purple), or PBS control (gray). Data are represented as individual values per BM donor. The cohort comprised 12 NSG recipient mice, where 3 different *Cdk6* ^+/+^ *CALR* ^*del52*^ BM donor mice were used: #1, #2, and #3, each of them transplanted into four NSG recipient mice, each of them receiving one treatment condition. Log‐transformed platelet counts were analyzed using a linear regression model in R, accounting for the donor relationship. Spleen‐to‐body‐weight ratios were analyzed using a beta regression model in R, accounting for the donor relationship. Pairwise statistical tests were performed with the emmeans package in R. P‐values were adjusted using the Tukey method for comparing a family of four estimates. *P < 0.05; **P < 0.01; ***P < 0.001; and ****P < 0.0001. **(C)** Donor chimerism. Donor BM (CD45.2^+^) total cell counts of BM and SP (left) and percentages in PB (right) of NSG recipients. **(D)** Flow cytometric analysis of hematopoietic stem and progenitor cell (HSPC) subpopulations in BM (left) and SP (right) of NSG recipient mice. HSPC subsets comprise hematopoietic stem cell (HSC)/MPP1, MPP2, and MPP3/4 cell populations. **(E)** Annexin‐V/DAPI apoptosis staining showing percentages of donor BM (CD45.2^+^) cells in NSG recipient BM (left) and SP (right). **(F)** Representative pie‐chart of Annexin‐V/DAPI apoptosis staining on BM HSC/MPP1 (from L^−^K^+^CD86^+^) cells from NSG recipients. **(G)** Annexin‐V/DAPI apoptosis staining showing percentages of host BM (CD45.1^+^) (left) and host HSC/MPP1 BM cells (right) in NSG recipient mice. **(H)** Summary bar plot comparing percentages of living cells in host (CD45.1^+^) and donor (CD45.2^+^) BM upon treatments determined by Annexin‐V/DAPI apoptosis staining in NSG recipient mice. Error bars represent mean±SD. *n* = 3 per condition. *P < 0.05; **P < 0.01 by ordinary one‐way analysis of variance (ANOVA) followed by Tukey's multiple comparison test. LSK, Lin^−^ c‐Kit^+^ Sca‐1^+^.

To evaluate the *in vivo* efficacy of CDK6 kinase inhibition combined with pegIFNα stimulation, we transplanted the total BM of *Cdk6* ^+/+^ *CALR* ^*del52*^ mice into NSG recipients and treated the mice with palbociclib daily and pegIFNα weekly over 3 weeks, starting at week 9 post‐transplantation when the disease was fully developed (Figure 4A). Palbociclib/pegIFNα combination treatment, as well as single pegIFNα, showed a significant reduction of the MPN disease phenotype compared to controls as measured by reduced SW and platelet counts (Figure 4B). No signs of fibrosis have been observed in the different groups. Mice treated with both drugs displayed significantly fewer donor cells (CD45.2^+^) in the PB circulation, while donor cells in BM and spleen were only slightly reduced (Figure 4C). Similar to the observed effects of pIpC on *Cdk6* ^−/−^ *CALR* ^*del52*^ mice, combinatorial treatment with palbociclib/pegIFNα, when compared to controls, significantly reduced HSC/MPP1 and CD41 cell populations while increasing MPP2 cells in the spleen, with no major differences detected in the BM (Figures 4D and S4A). Moreover, when compared to pegIFNα alone, treatment with palbociclib/pegIFNα showed a significant reduction of donor HSC/MPP1 cells in the BM and spleen. No significant differences between single pegIFNα‐treated and control samples were observed across all HSPC populations (Figure 4D).

Consistent with the increased apoptosis seen in pIpC‐treated mice lacking *Cdk6*, we determined elevated apoptosis in the donor (CD45.2^+^) cells in both the BM and spleen of palbociclib/pegIFNα‐treated mice compared to controls (Figure 4E). Importantly, only mice receiving combinatorial treatment displayed significantly increased apoptosis in BM HSC/MPP1 cells compared to controls, while no effects on this cell population were observed using single treatments (Figure 4F,G). Strikingly, these effects were restricted to malignant donor cells and were not observed in benign host BM cells (CD45.1^+^) of these mice (Figure 4G,H). These findings underline that inhibition of CDK6 improves the treatment of *CALR*‐driven MPN with pegIFNα by specifically inducing cell death.

### Transcriptomic profiles of MPN patients reveal CDK6‐IFNAR1 inverse correlation

Studies suggest that MPN patients respond differently to IFN treatment dependent on their mutation status.^23^, ^61^, ^62^ To further investigate this observation, we analyzed RNA‐Seq data of a cohort comprising 230 human patients (64 controls, 86 *JAK2* ^*V617F*^, 39 *CALR*‐mutant, 41 co‐mutations with *ASXL1*, *MPL*, *CBF*, *SRSF2*, *TET2*, *DNMT3*, and *EZH2*).^43^

**Figure 5:**
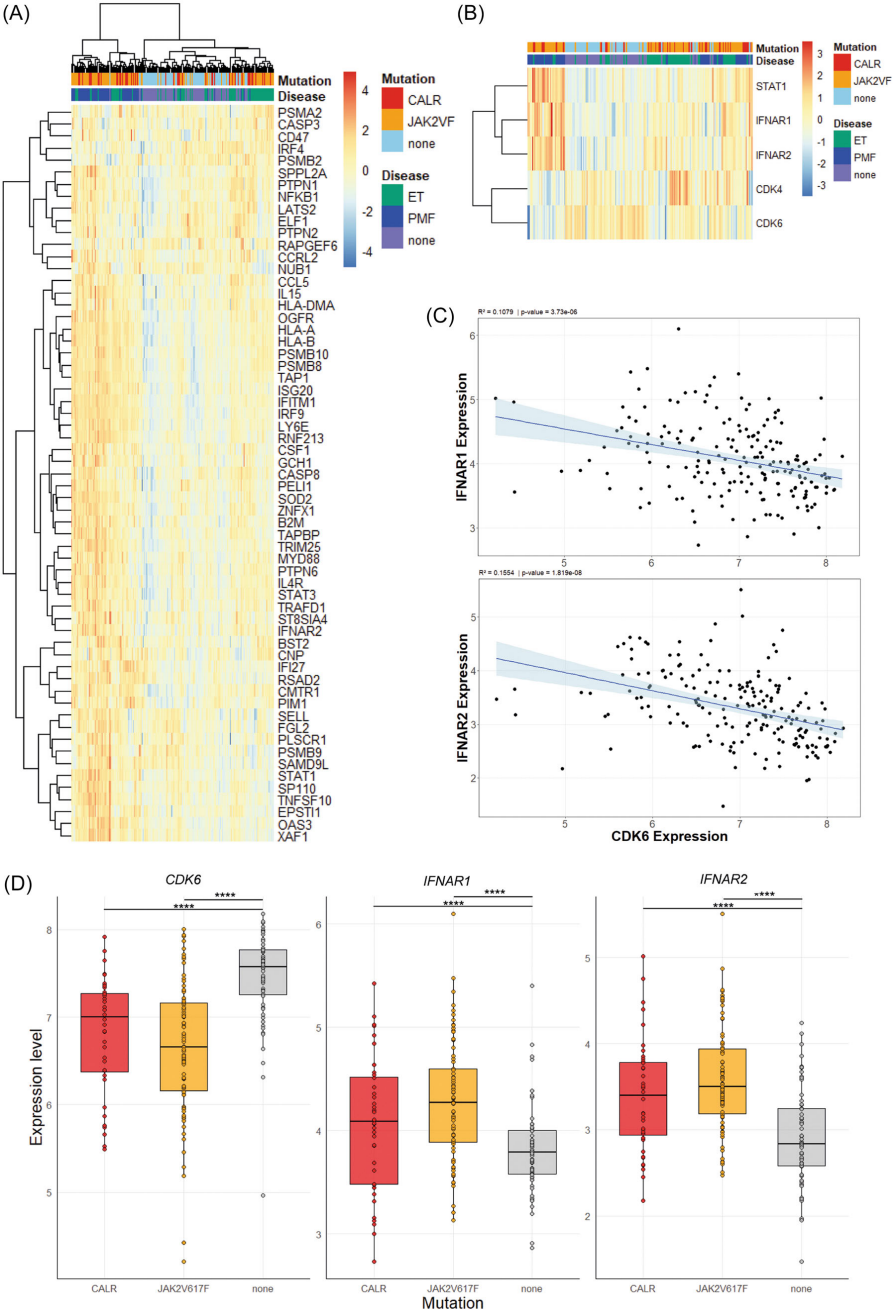
***CDK6* expression negatively correlates with interferon‐alpha receptor (IFNAR) gene expression in bone marrow (BM) and peripheral blood (PB) from myeloproliferative neoplasm (MPN) patients. (A)** Heatmaps showing RNA‐Seq data of BM and PB samples from a 230‐patient cohort comprising 64 controls without mutation or disease, 86 *JAK2* ^*V617F*^ mutations, and 39 *CALR* mutations. The heatmap was generated using the pheatmap package in R‐Studio. Plotted are log2CPM normalized counts that have been scaled across samples. Clustering was done using the ward. D2 clustering method. RNA‐Seq gene signature of interferon‐alpha and interferon‐gamma signaling pathways obtained from GSEA of *Cdk6* ^−/−^ *CALR* ^*del52*^ versus *Cdk6* ^+/+^ *CALR* ^*del52*^ MkPs was used to determine this signature in MPN patients and controls. **(B)** *CDK4*, *CDK6*, *STAT1*, *IFNAR2*, and *IFNAR1* were clustered and depicted as genes of interest. Clustered groups are depicted as *CALR* (red), *JAK2* ^*V617F*^ (orange) patients, either harboring essential thrombocythemia (ET) (green) or primary myelofibrosis (PMF) (blue) disease, and controls with no mutations (sky blue), which were clustered as having no disease (gray). **(C)** Scatterplot showing a linear regression analysis of a 230‐patient cohort negatively correlating *CDK6* levels in BM and PB of patients with *IFNAR1* and *IFNAR2* expression. The graphics and linear regression were performed using R‐Studio. **(D)** Bar plots of 39 *CALR*, 86 *JAK2* ^*V617F*^, and 64 control patient samples from BM and PB, clustered according to their mutation and irrespective of disease, showing *CDK6*, *IFNAR1*, and *IFNAR2* expression levels. The plots were generated using the ggplot2 package in R‐Studio. *P < 0.05; **P < 0.01; ***P < 0.001; and ****P < 0.0001 by ordinary one‐way analysis of variance (ANOVA) followed by Tukey's multiple comparison test.

We opted to identify if MPN patients show transcriptional changes of the genes related to IFN‐associated pathways, which were affected by the presence of CDK6. Interestingly, analysis of RNA‐Seq profiles within BM and PB of MPN patients exhibited highly upregulated IFN gene patterns compared to healthy controls. These effects were more frequently seen in PMF than in ET patients (Figures 5A and S5A,B). While no differences in the expression IFN‐related genes were observed between *JAK2* ^*V617F+*^ and *CALR*‐mutated patients in the expression of the IFN‐related genes (Figure S5C), the upregulation of IFN‐associated pathways was identified as a general feature of MPN malignancy, which is in line with recent reports.^63^, ^64^, ^65^, ^66^

In general, *STAT1*, *IFNAR1*, and *IFNAR2* mRNA expression levels were higher in PMF than in ET patients compared to controls. The analysis of *JAK2* ^*V617F*^ and *CALR*‐mutated PMF or ET patients revealed that patients with low *CDK6* levels have high levels of *IFNAR1*, *IFNAR2*, and *STAT1* in PB or BM cells and vice versa, underlining an inverse CDK6‐IFNAR1 regulation (Figure 5B). We observed a significant negative correlation of *IFNAR1* (*R* ^2^ = 0.1079) as well as *IFNAR2* (*R* ^2^ = 0.1554) and *CDK6* gene expression independent of mutation or disease status (Figure 5C,D). Moreover, upon distinguishing *CALR* Type 1 (22) and *CALR* Type 2 (17) patient transcriptomic data, we found that only *IFNAR2* gene expression remained significantly different in *CALR* Type 1 patients and that no differences between *CALR* types were observed (Figure S5D), which might be due to low sample sizes upon distinction. These data led to the hypothesis that patients with low CDK6 levels could be more susceptible to IFNα therapy, at least partially due to high IFNAR levels.

### CDK4/6 inhibition allows dose reduction of pegIFNα in MPN patient samples

Treatment of MPN patients with IFNα has been a successful approach for many decades; however, patients often develop multiple side effects, and some do not respond as well as others.^17^, ^22^, ^62^ We set out to test if we can lower pegIFNα concentrations when combined with CDK4/6 inhibition. First, a series of palbociclib and pegIFNα concentrations was tested on murine HPC^LSK^ cell lines expressing *CALR* ^*del52*^ to determine optimal effective concentrations for inducing apoptosis and CD41 expression as a marker of MK‐differentiation. Lower combined concentrations were similarly effective in inducing apoptosis and CD41 expression compared to commonly used dosages of 200 nM palbociclib^25^, ^33^ and 600 ng pegIFNα^67^ (data not shown), prompting us to use reduced dosages for further experiments. Moreover, we show that palbociclib and pegIFNα acted synergistically in HPC^LSK^ cells harboring a *CALR* mutation (Figure S6A).

**Figure 6:**
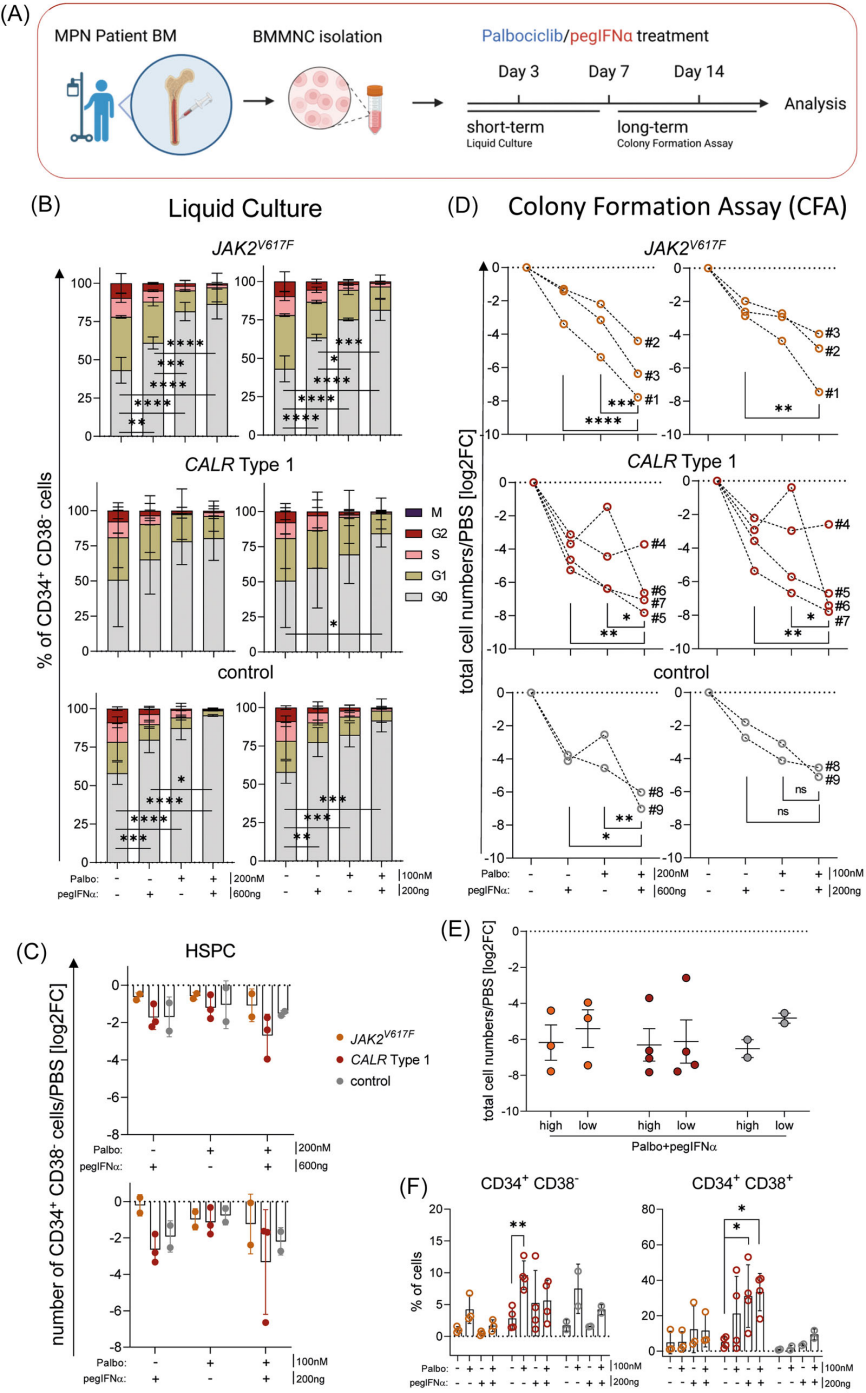
***Cdk6* inhibition allows reduction of pegylated interferon‐α (pegIFNα) for myeloproliferative neoplasm (MPN) patient‐derived bone marrow mononuclear cell (BMMNC) treatment. (A)** Experimental scheme: patient‐derived BM of 10 patients (3 *JAK2* ^*V617F*^–2 ET/1 PMF, 4 *CALR* Type 1–2 ET/2 PMF, and 3 lymphoma control–1 NHL/2 lymphoma) was isolated to receive BMMNCs, which were treated with 200 nM palbociclib, 600 ng pegIFNα, in combination or phosphate‐buffered saline (PBS) control. Additionally, lowered doses of 100 nM palbociclib, 200 ng pegIFNα, and respective combinations were used. Treatments were administered short‐term in liquid culture (3 days) or long‐term using a colony formation assay (7–14 days). Created in BioRender. Kollmann, K. (2026) https://BioRender.com/2302srg **(B)** Patient‐derived BMMNCs were cultured in liquid culture medium for 3 days and then assessed for cell cycle activity upon treatments. Graphs represent cell cycle stainings of HSPCs (CD34^+^ CD38^‐^) using Ki67/DAPI from short‐term treatment of *JAK2* ^*V617F*^ (top), *CALR* Type 1 (middle), and control (bottom) patients using 200 nM palbociclib, 600 ng pegIFNα, in combination or PBS control (left) and in lower dosages with 100 nM palbociclib, 200 ng pegIFNα, in combination or PBS control (right). *P < 0.05; **P < 0.01; ***P < 0.001; and ****P < 0.0001 by two‐way analysis of variance (ANOVA) followed by Tukey's multiple comparison test. **(C)** Summary bar blot showing log2 fold‐changes of HSPC (CD34^+^ CD38^−^) numbers obtained from short‐term liquid culture experiments. **(D)** BMMNCs from MPN and control patients were cultured in fully supplemented methylcellulose for 7–14 days. Graphs represent colony formation assays of *JAK2* ^*V617F*^ (orange, Patient #1, #2, and #3), *CALR* Type 1 (red, Patient #4, #5, #6, and #7), and control (gray, Patient #8 and #9) patients using 200 nM palbociclib, 600 ng pegIFNα, in combination or PBS control (left) and in lower dosages with 100 nM palbociclib, 200 ng pegIFNα, in combination or PBS control (right). Data represent calculated log2 fold‐change of total cell numbers with treatment conditions normalized to PBS controls within BMMNCs in fully supplemented methylcellulose, obtained by flow cytometry. Each line represents one patient's BM sample, each dot a treatment condition within the sample. Statistical analysis was performed using a mixed‐effects model as described in the methods. *P < 0.05; **P < 0.01; ***P < 0.001; and ****P < 0.0001. **(E)** Summary dot blot showing log2 fold‐changes of total cell numbers from *JAK2* ^*V617F*^ (orange), *CALR* Type 1 (red), and control (gray) patients using 200 nM palbociclib, 600 ng pegIFNα, in combination over PBS control (high) and 100 nM palbociclib, 200 ng pegIFNα, in combination over PBS control (low) after colony formation assays. Each dot represents a patient sample in a condition. **(F)** Flow cytometric analysis of colonies from patient‐derived BMMNCs from *JAK2* ^*V617F*^ (orange), *CALR* Type 1 (red), and control (gray) patients using 100 nM palbociclib, 200 ng pegIFNα, in combination or PBS control. Left: Percentages of HSPC (CD34^+^ CD38^−^) populations within colonies. Right: Percentages of HPC (CD34^+^ CD38^+^) populations within colonies. Each dot represents a patient sample in a condition. The treatment prior to analysis was done for 14 days. Error bars represent mean ± SD. *n* ≥ 2 per condition. *P < 0.05; **P < 0.01; ***P < 0.001; and ****P < 0.0001 by two‐way ANOVA followed by Tukey's multiple comparison test.

Isolated human BMMNCs from *JAK2* ^*V617F*^, and *CALR* Type 1 MPN patients (PMF or ET) and control patients were used for suspension and CFAs to elucidate the effects of single and combinatorial treatments with palbociclib and pegIFNα to confirm murine data in a patient‐relevant setting (Figure 6A and Tables S2 and S3). In a short‐term liquid culture of BMMNCs, HSPCs receiving combinatorial treatment remained most quiescent (G0‐phase), indicated by cell cycle arrest (Figure 6B).^61^ Interestingly, lower dosages of palbociclib and pegIFNα yielded similar effects compared to the higher concentrations when analyzing HSPC numbers (CD34^+^ CD38^−^ cells), especially in *CALR*‐mutant cells (Figures 6C and S6B). In contrast to MPN samples, revealing no differences across concentrations, we detected a reduced HSPC cell cycle arrest at lower treatment dosages in the control samples.

To analyze prolonged effects on colony‐forming MPN cells in patient samples, we performed CFAs. Total cell numbers were reduced more than sixfold in MPN patient samples when treated with palbociclib/pegIFNα (Figures 6D, and S6C,D,F). Of note, the low‐dose condition elicited an effect comparable to the higher dose in *CALR*‐mutated patient samples, indicating that maximal therapeutic impact can be achieved even at reduced concentrations. In contrast, control samples exhibited a slight reduction in cell numbers under the same low‐dose treatment, suggesting increased sensitivity of non‐mutant cells at higher doses (Figures 6E and S6D). This differential response highlights a potential therapeutic window, wherein *CALR*‐mutant cells can be effectively targeted with minimal impact on healthy hematopoietic cells. The percentages of HSPCs within cell colonies were increased among colonies in palbociclib‐only conditions, potentially due to their protective state and increased fitness as reported recently.^33^, ^39^ This effect was not observed in combination‐treated colonies of the *CALR*‐mutated patients, as these led not only to reduced living cells but also induced differentiation into CD34^+^ CD38^+^ HPCs and, as expected, CD41^+^ CD42^+^ MKs (Figures 6F and S6E). As differences between the *CALR‐*mutant types have been proposed recently,^68^ we performed similar assays with BMMNCs from *CALR* Type 2 MPN patients (PMF or ET) (Figure S6F–I). Similar effects to cells harboring the *CALR* Type 1 mutation have been observed, highlighting no relevant difference between the different *CALR* types for our treatment strategy. To determine if the combinatory treatment also affects the mutational burden, we performed next‐generation sequencing (NGS) of MPN patient BMMNCs after treatment in a CFA. A significant reduction in total colony numbers in the combinatorial treatment compared to the single treatments has been observed (Figure S6F). Although a CFA setting only provides a short and quite limited time period to alter the allelic burden of MPN cells, as described using pegIFNα,^39^, ^69^ we observed indications of reduced mutational burden in *CALR* patient samples mediated by palbociclib, which were not observed in *JAK2*‐mutated cells (Figure S6I). To further evaluate whether the combinatory approach of CDK6 inhibition and pegIFNα preferentially targets malignant cells, vividly mimicking a mutational burden setting, we performed an *in vitro* co‐cultivation assay of *CALR* ^*del52*^ or *JAK2* ^*V617F*^ together with wild‐type HPC^LSK^ cell lines in a proliferative and MK‐differentiation environment (Figure S6J). Similar to the observations found *in vivo* (Figure 4), palbociclib/pegIFNα combination significantly reduced the percentage of *CALR* ^*del52*^ or *JAK2* ^*V617F*^ expressing cells compared to controls and single treatments upon 6 days of culture, which was not observed for wild‐type HPC^LSK^ cells (Figure S6K,L). When differentiating *CALR* ^*del52*^ or *JAK2* ^*V617F*^ expressing cells into MKs in the presence of TPO, we again observed the strongest effect on MK‐differentiation using the combinatorial approach (Figure S6M–O), highlighting that palbociclib and pegIFNα combination treatment favors cell death of malignant HSPCs and leads to enhanced MK‐differentiation.

To understand the correlation between CDK6 levels and treatment response, we determined CDK6 protein levels in patient BM‐derived CD34^+^ CD38^−^ cells (HSPCs). CDK6 protein levels significantly correlated with the effectiveness of palbociclib/pegIFNα treatment, reducing HSPC numbers (Figure S6P). MPN patient HSPCs with low CDK6 levels responded better to drug‐induced effects on malignant cells. We then sought to determine if these CDK6 protein levels correlate with IFNAR expression, similar to the observations found in MPN patient transcriptomic data. Upon CDK6 inhibition, we determined increased IFNAR1 but not IFNAR2 surface expression on MPN mutant cells. This was also reflected by CDK6‐IFNAR protein interplay, where IFNAR1 negatively but IFNAR2 positively correlated with intracellular CDK6 protein levels of MPN patient BMMNCs (Figure S6Q). These findings highlight that CDK6 might not only repress IFNAR1 and IFNAR2 transcriptionally but might also differentially regulate their protein levels in a context‐dependent manner.

Our data propose a novel therapeutic strategy to treat MPN patients. CDK6 inhibition sensitizes *CALR* ^*del52*^ cells to IFNα treatment by modulating IFNα‐receptor expression and deregulating IFN ‐response pathways. CDK6 inhibition in combination with IFNα treatment allows the reduction of IFNα concentrations without losing responsiveness of the malignant cells. Moreover, patient subgroups with high CDK6 levels could preferentially benefit from the effects of palbociclib to increase IFNAR1 and thus make them more susceptible to pegIFNα therapy.

## DISCUSSION

We identify CDK6 as a crucial negative regulator of IFN signaling in *CALR*‐mutant MPN and demonstrate that targeting CDK6 significantly sensitizes malignant cells to IFNα therapy. Genetic deletion of *Cdk6* in *CALR* ^*del52*^ knockin mice^42^ mitigated MPN disease phenotypes, including splenomegaly, thrombocytosis, and HSPC expansion. This is in line with previous studies on *JAK2* ^*V617F*^‐driven mouse models, where CDK6 inhibition and deletion significantly diminished PV‐ and PMF‐like MPN disease phenotypes.^39^, ^40^, ^70^ The effects of CDK6 inactivation on *CALR*‐mutant cells persisted after transplantation into NSG recipients, suggesting a cell‐intrinsic mechanism. Transcriptomic analyses of MkPs revealed significant enrichment of IFN‐associated pathways, including an increase in *IFNAR1* and *IFNAR2* gene expression, and caspase‐mediated apoptosis genes, only in CDK6‐deficient cells. Opposing to previously published studies of *JAK2* ^*V617F*^‐mutated MPNs highlighting a strong kinase independent function of CDK6,^39^ we propose that the effects described here in *CALR‐*mutated cells are rather kinase‐dependent.^31^ As signaling downstream of *JAK2* ^*V617F*^ and mutant *CALR*, as well as the phenotype of the mouse models, shows high discrepancies, a detailed side‐by‐side analysis of the same cell populations with CDK6 loss would give more insights into CDK6 functions in the two disease subsets. While *CALR* mutation alone indicated suppression of these gene signatures, the loss of *Cdk6* reversed this effect, indicating that CDK6 acts as a brake on IFN‐mediated gene expression and cell death. Importantly, this IFN signature was detected in BM‐derived MkPs as opposed to spleen‐derived MkPs. This tissue‐specific difference is likely explained by the fact that basal type I IFN signaling is physiologically high in BM of MPNs,^71^, ^72^ while the spleen, as an extramedullary organ in MPN, provides a vastly different milieu for migrated malignant HSPCs and their progeny. How splenic myeloid and MkPs differ from their BM descendants remains poorly understood.^73^, ^74^, ^75^ Nonetheless, the result of our findings could be such that de‐repressing IFN pathway genes by the absence of CDK6 become transcriptionally detectable only in the BM, whereas splenic MkPs might be exposed to insufficient tonic IFN stimulation to activate these programs despite CDK6 loss. Concordantly, studies demonstrated that CDK6 inactivation triggered inflammatory cellular mechanisms in *JAK2* ^*V617F*^ MPN mouse models.^39^, ^70^

Induction of inflammatory pathways via pIpC selectively induced apoptosis in *CALR* ^*del52*^ HSPCs lacking *Cdk6*, particularly in HSC/MPP1 populations, a phenotype not observed in *CALR* ^*del52*^ or non‐mutant counterparts. pIpC primarily activates TLR3 signaling, triggering endogenous type I Interferon production, which in turn upregulates IFNAR1 to further elicit downstream immune responses, such as apoptosis.^76^, ^77^ IFN‐mediated apoptosis in HSPCs depends on IFNAR and STAT1 signaling and supports our finding that loss of CDK6 sensitizes cells to such stimuli.^52^, ^53^, ^77^ CDK6 modulates the expression of SOCS1 and SOCS3, which are key negative feedback regulators of IFNAR signaling in T cells, suggesting a broader immunoregulatory role.^78^ Similarly, CDK6 influences the activity of protein tyrosine phosphatases (PTPNs), especially *PTPN1* (coding for PTPB1), which is not only involved in type 1 interferon responses^79^, ^80^, ^81^ but also revealed to be highly upregulated in our MkP *Cdk6*‐ablated transcriptomic data. Thus, the absence of CDK6 may increase PTPB1 activity or induce *PTPN1* expression, allowing prolonged and amplified interferon responses, ultimately shifting the cellular fate from survival to apoptosis in malignant HSPCs. In addition, the analysis of the CDK6 interactome and phospho‐targets in mutant *CALR* models would help to clarify the influence of CDK6 on IFN signaling by protein‐protein interactions.

pegIFNα is a clinically approved therapy for PV.^82^ It is worth noting that pegIFNα is capable of inducing molecular remissions in a subset of MPN patients, although its efficacy is often variable, and some of the patients suffer from inflammatory toxicity.^22^ CDK6 inhibition appears to sensitize *CALR*‐mutant HSPCs to interferon signaling by increasing IFNAR expression and priming the cells for stronger downstream responses. The used combination of CDK6 inhibition with pegIFNα may tilt the balance toward apoptosis in malignant cells, allowing a mechanistic basis for the observed synergy.

*JAK2* and *CALR* mutations have been associated with differing responses to IFN therapy. In fact, *JAK2*‐mutant MPNs often show stronger clinical responses to pegIFNα than *CALR*‐mutant ones, accompanied by higher STAT1 activation and IFNAR expression in patients.^23^, ^83^ To better understand disease‐specific and mutation‐associated signaling states, we analyzed previously published RNA‐Seq data from MPN BM and PB samples.^43^ We observed elevated expression of *IFNAR1*, *IFNAR2*, and *STAT1*, along with our IFN‐associated MkP gene signature, in PMF compared to ET samples. However, no significant differences in the IFN‐related markers were observed between *JAK2* ^*V617F*^ and *CALR*‐mutant patients, independent of Type 1 or Type 2, suggesting that other factors may influence IFN signaling and inflammation‐associated pathways. We identified CDK6 as one such factor, given its significant negative correlation with elevated *IFNAR1* and *IFNAR2* expression. *CDK6* expression was significantly reduced in MPN samples compared to healthy controls, which contrasts with findings of MPN granulocytes harboring higher *CDK6* expression.^40^ We observed slightly elevated CDK6 protein levels in the HSCs of both PMF and ET patients, juxtaposed to our clinical transcriptomic data. These findings underline the fact that *CDK6* expression can be dynamic and cell state‐dependent, as previous studies described the oscillatory expression of *CDK6* in various HSC subpopulations.^26^ In addition, these data suggest that a reduction in CDK6 might render malignant cells more susceptible to IFN signaling. CDK6 inhibition may therefore enhance this primed state by further elevating IFN responsiveness.

When considering the pegIFNα/palbociclib combination in the treatment of patients, one important aspect is toxicity. Therefore, we were interested in showing combination effects that effectively block the expansion of MPN cells without reducing normal BM cells in the same way, providing a therapeutic window. Indeed, we identified a dose combination of palbociclib and pegIFNα that preserved efficacy in patient‐derived MPN cells, while reduced cytotoxicity against normal healthy cells was observed. Although the full‐dose combination therapy affected non‐MPN BM cells, lowering the dose moderately reduced apoptosis in control samples while maintaining similar anti‐clonal effects in MPN cells. This suggests that malignant cells may remain susceptible at reduced drug concentrations due to their higher proliferative potential and increased IFNAR‐mediated signaling. As a result, it may be possible to selectively target the malignant clone while limiting toxicity to normal hematopoiesis. To evaluate whether the combination selectively affects the malignant compartment, we assessed changes in the mutant allele burden following single and combinatory treatments using NGS. While indications of a CDK6 inhibition effect on reducing the mutational burden are recognized, we did not conclude consistent changes, particularly in the combinatorial treatments. This was expected due to a short treatment period and small sample size, and is in line with observations in *JAK2* ^*V617F*^‐mutant cells upon pegIFNα treatment *in vitro*.^39^, ^69^ However, when using a co‐culture system of malignant and benign HPC^LSK^ cell lines in a proliferative setting, our combinatory approach seemed to favorably affect mutated cells with reduced effects on wild‐type cells. These findings underline that a prolonged treatment and a large‐scale cohort would be necessary to determine substantial effects on mutational burden upon palbociclib and pegIFNα combination therapy in patients.

Palbociclib and IFNα treatment are known to drive cell differentiation,^55^, ^84^, ^85^ as validated in our assays. Combinatory treatments with palbociclib and pegIFNα led to significantly increased MK‐lineage differentiation compared to single treatments in our *in vitro* and *in vivo* experiments, while depleting the stem cell pool. Overall, pegIFNα had the weakest effect on MK‐differentiation compared to palbociclib or combinatorial treatment.

After determining intracellular CDK6 protein levels in MPN patient BMMNCs, we found that patients with low CDK6 levels in their CD34^+^ CD38^−^ HSPCs showed the most robust responses to the combinatorial treatment. As CD34^+^ CD38^−^ expressions of primary patient cells can shift upon *in vitro* cultivation and may not fully capture long‐term repopulating HSCs,^86^ our findings relate primarily to enriched HSPCs. Future *in vivo* studies will help clarify how the effects of combinatory treatment extend to the most primitive stem cell populations. We next investigated the post‐transcriptional relationship between CDK6 and IFNAR expression. CDK6 protein abundance negatively correlated with IFNAR1 surface expression on patient‐derived BMMNCs, mirroring the relationship observed in the patient transcriptomic data, whereas no such association was detected for IFNAR2. This pattern suggests a selective coupling between CDK6 and IFNAR1‐driven IFN responsiveness, reinforcing the presumption that IFNAR1 rather than IFNAR2 is the dynamically regulated determinant of the heterodimeric complex for IFNα sensitivity in these cells.^87^, ^88^, ^89^

Since hematologic side effects such as neutropenia and anemia are closely linked to CDK6 activity,^90^, ^91^ future development of CDK6‐selective degraders or PROTACs may enhance therapeutic precision and reduce off‐target effects compared to broader CDK4/6 inhibitors.^92^, ^93^, ^94^ Furthermore, the underlying mechanism of cell death, whether mediated through STAT1‐caspase signaling, RIPK1‐dependent necroptosis, or other interferon‐induced pathways, remains to be elucidated.^95^, ^96^, ^97^, ^98^, ^99^ Gaining deeper insight into these processes could help refine therapeutic strategies and enhance potential patient selection.

In conclusion, we propose a novel molecular mechanism in which CDK6 functions as a central gatekeeper of IFN signaling in both *CALR*‐ and *JAK2*‐mutant MPNs. We introduce a potential therapeutic option where CDK4/6 inhibition sensitizes malignant HSPCs to IFNα‐induced apoptosis, even at reduced drug concentrations. The low‐dose regimen achieved equivalent therapeutic efficacy in targeting malignant HSPC clones compared to the high‐dose treatment, while slightly reducing toxicity in controls. This therapeutic strategy may offer mutation‐independent potential to minimize treatment‐related side effects on normal hematopoiesis.

## AUTHOR CONTRIBUTIONS

Brian Ringhofer and Wolfram Polzer designed, and Brian Ringhofer, Wolfram Polzer, Eszter Doma, Belinda S. Maw, Elisabeth Gamper, and Michaela Prchal‐Murphy conducted experiments. Brian Ringhofer, Wolfram Polzer, and Jonatan Kendler analyzed the data. Brian Ringhofer, Sebastian Kollmann, and Thorsten Klampfl performed analyses of bioinformatics. Eva Zebedin‐Brandl, Juan Li, Anthony R. Green, Maria‐Theresa Krauth, Emir Hadzijusufovic, Daniel Ivanov, Peter Valent, and Gregor Hoermann provided reagents, materials, and transcriptomic data. Veronika Sexl and Karoline Kollmann were involved in the conception and design of the study and contributed essential material. Karoline Kollmann designed and supervised experiments. Karoline Kollmann designed and supervised the study, and Brian Ringhofer and Karoline Kollmann wrote the manuscript.

## CONFLICT OF INTEREST STATEMENT

The authors declare no conflicts of interest.

## FUNDING

This research was funded in whole or in part by the Austrian Science Fund (FWF) SFB‐F6107 (V.S.) and the Austrian Science Fund (FWF) P31773 (K.K.) and grant DOI 10.55776/PIN1612324. This research was funded in part by the Gesellschaft für Forschungsförderung Niederösterreich m.b.H. (GFF, LSC20‐004). For open access purposes, the author has applied a CC BY public copyright license to any author‐accepted manuscript version arising from this submission. Open Access funding was provided by Veterinarmedizinische Universitat Wien/KEMÖ.

## Supporting information

Supporting Information.

## Data Availability

The data that support the findings of this study are openly available in ArrayExpress at https://www.ebi.ac.uk/biostudies/arrayexpress/studies/E-MTAB-15404?key=dde031d5-a6c4-44ce-9032-668ddbc14f89, reference number E‐MTAB‐15404.
