# Supplementary material for: CDK6 inactivation counteracts CALR‐mutant‐induced MPN evolution and sensitizes MPN stem cells to interferon‐α treatment
Source: Hemasphere. 2026 May 12;10(5):e70363. doi: 10.1002/hem3.70363 (PMC13162134; doi:10.1002/hem3.70363)
Supplement: Supplementary file 1 — Supporting Information. [file HEM3-10-e70363-s001.docx]

**Supplementary Information**

**CDK6 inactivation counteracts CALR-mutant-induced MPN expansion and sensitizes MPN stem cells to Interferon-α treatment**

**SUPPLEMENTARY MATERIALS AND METHODS**

**HPC^LSK^ cell line establishment**

HPC^LSK^ cell lines were established, maintained and cultured as previously described.^1^ To generate MPN-associated *CALR*-mutant HPC^LSK^ cell lines, Platinum-E (plat-E) cell lines maintained in DMEM medium supplemented with 10% fetal calf serum (FCS, Capricorn Scientific) were used for retroviral transfection. Plat-E cells were separately transfected with the following retroviral vectors: pMSCV-CALRdel52-IRES-GFP using Turbofect (Thermo Scientific) according to the manufacturer’s instruction. After 24h, DMEM medium was changed to IMDM culture media supplemented with 5% FCS, 100 U/ml penicillin/streptomycin (P/S) and 15 mM 1-thiolglycerol (MTG; Sigma-Aldrich) and incubated for 24h. Viral supernatants were harvested, filtered (0.45 μm) and supplemented with 4 μg/ml polybrene, 2% stem cell factor (SCF) and 12.5 ng/ml IL-6 (Peprotech) for HPC^LSK^ cell transfection. After one week of retroviral transduction, GFP-positive HPC^LSK^ cells were sorted using Cytoflex (Beckman Coulter).

**HPC^LSK^ cell line culture**

HPC^LSK^ cell lines were cultured as previously described.^1^ Briefly, HPC^LSK^ cells were seeded on 1% agarose coated culture plates, in IMDM culture media supplemented with 12.5 ng/ml IL-6 (PeproTech), 2% SCF in a 5% CO_2_ humidified incubator. Cells were continuously kept at a density between 0.5-1x10^6^ cells/ml. The virus packaging cell lines Platinum-E (Plat-E, Cell Biolabs) were kept in DMEM (Sigma) supplemented with 10% FCS and P/S.

**Flow Cytometry**

***Surface staining***

Bone marrow (BM) cells from mice were isolated by crushing of femora and tibiae in phosphate-buffered saline (PBS) using a mortar and pestle. The cell suspension was filtered through a 70 µm cell strainer and washed once in PBS. Cells were stained on ice for 30 min in PBS. For characterization of the HSC compartment and flow cytometer-sorting of LSK (Lineage^-^ (Lin^-^) c-Kit^+^ sca-1^+^), HSC/MPP1 (Lin^-^ c-Kit^+^ sca-1^+^ CD48^-^ CD150^+^) or MkP (Lin^-^ c-kit^+^ sca-1^-^ CD41^+^ CD150^+^) cells derived from BM of *Cdk6^+/+^* *CALR^+/+^*, *Cdk6^-/-^* *CALR^+/+^*,*Cdk6^+/+^* *CALR^del52^* and *Cdk6^-/-^* *CALR^del52^* mice, antibodies directed against Lineage markers (TER119, CD11b, Gr-1, CD19, CD3)-APC/Cy7, Sca1-PE/Cy7, c-Kit-PE-Cy5, CD150-BV510, CD48-PE ad CD41-APC were used. To detect IFNAR1 levels of *Cdk6^+/+^* *CALR^+/+^*, *Cdk6^-/-^* *CALR^+/+^*, *Cdk6^+/+^* *CALR^del52^* and *Cdk6^-/-^* *CALR^del52^* BM cells and after *in vitro* assays, samples were stained with antibodies directed against: Lineage markers-APC/Cy7, CD3-APC/Cy7, Sca1-PE/Cy7 (alternatively CD86-PE/Cy7), c-Kit-PE-Cy5, CD150-BV510, CD48-BV605, CD41-APC and IFNAR1-PE. For NSG-recipient mice transplanted with donor BM, BM and spleen (SP) were analyzed using: CD45.1-PE, CD45.2-BV650, Lineage antibodies-APC/Cy7, Sca1-PE/Cy7 (alternatively CD86-PE/Cy7), c-Kit-PE-Cy5, CD48-BV605 (alternatively PE), CD150-APC (alternatively BV510), CD41-FITC. Human patient samples were stained with antibodies against: CD34-PE, CD38-PE/Cy7, CD11b-PE/Cy5.5, CD42a-APC or CD41-APC/Cy7. All samples were acquired using Cytoflex (Beckman Coulter) or Cytoflex S (Beckman Coulter) flow cytometer. Data were analyzed using CytExpert. All cell sorting experiments were performed on a FACSAriaIII device or CytoflexSRT (Beckman Coulter).

***Cell cycle staining***

KI67/DAPI staining was performed as described previously.^2^ Cells were stained using antibodies directed against: Lineage markers-APC/Cy7, c-Kit-PE-Cy5, Sca1-PE/Cy7 or CD86-PE/Cy7, CD150-Biotin, Streptavidin-BV650, CD48-PE and CD41-APC. For NSG-recipient mice transplanted with donor BM, BM and SP were stained using: CD45.1-PE, CD45.2-APC/Cy7, Lineage antibodies-Biotin, Streptavidin-BV650, Sca1-PE/Cy7 or CD86-PE/Cy7, c-Kit-PE-Cy5, CD48-BV605, CD150-APC. Human patient samples were stained with CD34-PE and CD38-PE/Cy7 antibodies. Cell cycle was analyzed using KI67-FITC and 2 µg/ml DAPI in PBS.

***Annexin-V staining***

Cells were collected and stained using antibodies directed against: Lineage markers-APC/Cy7, c-Kit-PE-Cy5, Sca1-PE/Cy7 or CD86-PE/Cy7, CD150-Biotin, Streptavidin-BV650, CD48-PE and CD41-APC. For NSG-recipient mice transplanted with donor BM, BM and SP were stained using: CD45.1-PE, CD45.2-APC/Cy7, Lineage antibodies-Biotin, Streptavidin-BV650, Sca1-PE/Cy7 or CD86-PE/Cy7, c Kit-PE-Cy5, CD48-BV605, CD150-APC. Human patient samples were stained with CD34-PE and CD38-PE/Cy7 antibodies. All further steps were performed in Annexin-V Binding Buffer (1x). Cells were washed and subsequently incubated for 10-15 min at room temperature (RT) with Annexin-V-FITC. Cell cycle was analyzed with 1x Annexin-V Binding Buffer containing 2 µg/ml DAPI.

***In vitro* experiments**

***HSC single-cell culture***

Single HSC/MPP1 (Lin^-^ c-Kit^+^ sca-1^+^ CD48^-^ CD150^+^) cells from *Cdk6^+/+^* *CALR^+/+^*, *Cdk6^-/-^* *CALR^+/+^*, *Cdk6^+/+^* *CALR^del52^* and *Cdk6^-/-^* *CALR^del52^* mice were sorted into 96-well round bottom dishes using a CytoflexSRT cell sorter (Beckman Coulter) and cultured in StemSpan SFEM II Medium (StemCell Technologies) with 10% FCS, 100 U/ml penicillin/streptomycin (P/S), 2mM L-glutamine, 200mM beta-mercaptoethanol, 40ng/ml mouse IL-11 and 2.5% SCF. Cells were counted manually using an OLYMPUS IX71 inverted microscope and subsequently harvested on day 7, followed by flow cytometric counting and analysis.

***Colony Formation Assay (CFA)***

100 LSK (Lin^-^ c-kit^+^ sca-1^+^) cells from *Cdk6^+/+^* *CALR^del52^* mice were sorted into sterile polysterene tubes, centrifuged, washed with 1x PBS and cultured in mouse methylcellulose (R&D Systems) supplemented with 20 ng /ml IL-3, IL-7, GM-CSF (R&D Sytems), IL-6 (PeproTech), 0.5% SCF, 200 ng/ml holo-transferrin (Sigma-Aldrich), 10 µg/ml insulin (Sigma-Aldrich) and 5 U/ml erythropoietin (EPO; Johnson & Johnson). Cells were resuspended in 2ml of this media in 35mm dishes and incubated at 37°C, 5% CO_2_ for 7-14 days. Colonies were counted by using an OLYMPUS IX71 inverted microscope, harvested, and analyzed via flow cytometry.

***In vitro treatment of HPC^LSK^ with Palbociclib/ropegIFNα2b***

1x10^3^ HPC^LSK^ overexpressing pMSCV-CALRdel52-IRES-GFP (*CALR^del52^*) were cultured in 96-well plates in 100µl fully supplemented IMDM medium with various concentrations of palbociclib (PD-0332991; Pfizer) and pegIFNα (ropegIFNα2b). To determine optimal dosages and possibly effective lower dosages of combinatorial treatments, *CALR^del52^* HPC^LSK^s were titrated with 50, 75, 100, 150 and 200nM palbociclib and 50, 100, 200, 300, 400, 500 and 600ng pegIFNα and incubated at 37°C, 5% CO_2_. After 48h, living cell numbers and CD41 expression were measured by flow cytometry using CD41-APC antibody.

***MK-differentiation assay***

Bulk-sorted LSK (Lin^-^ c-Kit^+^ sca-1^+^) cells from *Cdk6^+/+^* *CALR^del52^* mice were sorted into 96-well round bottom dishes using a CytoflexSRT cell sorter (Beckman Coulter) and cultured in StemMACS^®^ HSC Expansion Medium XF (Miltenyi Biotec) with 100 U/ml penicillin/streptomycin (P/S), 2mM L-glutamine, 2.5% SCF, 12.5ng/ml IL-6 and 100ng/ml rmTPO. Cells were treated with 200nM palbociclib and 100U rmIFNα (Merck Millipore) for 7 days. Subsequently, cell morphology of MK cells was investigated using an OLYMPUS IX71 inverted microscope and harvested, followed by flow cytometric counting and analysis.

**Assessment of drug synergism**

Drug synergy and dose response matrices were evaluated and generated using the R (version 4.1.1) package Synergyfinder (version 3.12).^3^ Drug combination response matrices were generated from cell viability data using two biological replicates of murine HPC^LSK^ *CALR^del52^* cell lines. Cells were treated with palbociclib and pegIFNα. Raw viability values were normalized to the untreated control (set as 0% Inhibition) and the maximum signal (100% inhibition). For the evaluation of synergistic effects regarding the CD41 expression, the percentage of CD41 positive cells were normalized to the baseline CD41 expression (set as 0% CD41 expression) and the maximum signal (100% CD41 Expression). Synergy scores were calculated using the Zero Interaction Potency (ZIP) reference model.^4^

**Immunohistochemistry (IHC)**

To evaluate hemopathology, spleen and BM (sternum tissue) were collected and fixed in 4% neutral buffered formalin. Bones were demineralized using OSTEOSOFT^®^ (Sigma-Aldirch, Merck Millipor) for 48-72 hours. Spleens and demineralized bones were processed routinely, embedded in paraffin, sectioned at a thickness of 2µm and stained with Hematoxylin and Eosin (H&E). Histologic slides were then imaged using an OLYMPUS IX71 inverted microscope.

**Patient samples experiments**

***Intracellular staining - patient samples***

To analyze intracellular CDK6 levels of *JAK2^V617F^*, *CALR* Type 1, *CALR* Type 2 and control patients (listed in Supplementary Table S2 and Supplementary Table S3), we used 4x10^5^ BM cells obtained from the liquid culture experiment. After collection from liquid culture, cells were washed once with PBS and then stained with CD34 and CD38 (HSPC; CD34^+^ CD38^-^/HPC; CD34^+^ CD38^+^) surface markers for 30min on ice. Surface-stained cells were washed once with PBS and fixed by 2% paraformaldehyde (Aldrich)/PBS at 37 °C, for 10 min. Cells were then permeabilized with 90% ice cold methanol in PBS/2% FCS/0.2% Tween-20 (ROTH) for 30 min at 4 °C on ice. Multiple washing steps were performed with PBS/2% FBS/0.2% Tween-20. After washing, cells were incubated with antibodies on ice for one hour. Cells incubated with the CDK6 antibody were washed twice and then incubated with IgG-APC/Cy7 (H+L) on ice for 20 min. Cells were washed two times before analysis via flow cytometry.

***JAK2 and CALR genotyping in colonies derived from human patients using next generation sequencing (NGS)***

5x10^4^ BMMNCs from *JAK2^V617F^*, *CALR* Type 1, *CALR* Type 2 and control patients (listed in Supplementary Table S3) were cultured in fully supplemented human methylcellulose (MethoCult H4435 Enriched). Cells were resuspended in 2 ml of this media in 35mm dishes and incubated at 37°C, 5% CO_2_ for 7-14 days.^5^ Colonies were counted by using an OLYMPUS IX71 inverted microscope, harvested, analyzed via flow cytometry using CD34 and CD38 surface antibodies (Supplementary Table S1) and collected for DNA extraction using a QIAmp DNA Micro Kit (Qiagen). DNA was then used for PCR amplification using a touchdown protocol followed by standard cycling. Reactions were initiated with an initial denaturation at 95°C for 1min. This was followed by 10 touchdown cycles, each consisting of denaturation at 95°C for 15s and extension at 72°C for 30s. Subsequently, 29 amplification cycles were performed with denaturation at 95°C for 15s, annealing at 67°C for 15s and extension at 72°C for 30s. A final extension step was carried out at 72°C for 5min, after which reactions were held at 4°C.

The following primers were used to amplify DNA at the sites of mutations using PCR.

| Gene | Type | Direction | Sequence |
| --- | --- | --- | --- |
| *JAK2* | NGSgt | F1 | ACACGACGCTCTTCCGATCTGGACAACAGTCAAACAACAATTCTTT |
| *JAK2* | NGSgt_ | R1 | GTGTGCTCTTCCGATCTACTGACACCTAGCTGTGATC |
| *CALR* | NGSgt | F2 | ACACGACGCTCTTCCGATCTCCCTGAGGTGTGTGCTCTG |
| *CALR* | NGSgt | R2 | GTGTGCTCTTCCGATCTCTCTACAGCTCGTCCTTGGC |

The underlined sequences represent partial Truseq adapter sequences (Illumina). The JAK2 gene-specific primer sequences have originally been published by Kralovics et al*.*^6^ After amplification, the PCR products were purified using the QIAquick PCR Purification kit (Qiagen). The purified PCR products were further processed at the Next Generation Sequencing Facility at Vienna BioCenter Core Facilities (VBCF), member of the Vienna BioCenter (VBC), Austria, to generate sequencing-ready libraries. This was done by performing a second PCR with primers that bind the partial Illumina adapter sequences and introduce sample-specific barcodes as well as P5 and P7 sequences. The final libraries were pooled and sequenced on an Illumina MiSeq i100+ sequencer in 150 bp paired-end fashion.

The resulting sequencing reads were demultiplexed and analyzed for the presence of wildtype (WT) and mutant (MUT) sequences of the JAK2 and CALR genes. By design, read 1 sequences mapped to the plus strand of the DNA, read 2 sequences to the minus DNA strand. The reads were therefore searched for the presence of the corresponding target sequences, in particular: *JAK2* samples:

WT_seq_plus: TGGAGTATGTGTCTGTGGAGA

WT_seq_minus: TCTCCACAGACACATACTCCA

MUT_seq_plus: TGGAGTATGTTTCTGTGGAGA

MUT_seq_minus: TCTCCACAGAAACATACTCCA

*CALR* Type 1 samples:

WT_seq_plus: GGACGAGGAGCAGAGGCTTAAGGAGGA

WT_seq_minus: CCTCCTTGTCCTCTGCCTCCTCCTCCT

MUT_seq_plus: GGACGAGGAGCAGAGGACAAGGAGGAT

MUT_seq_minus: CCTCCTTGTCCTCTGCTCCTCGTCCTG

*CALR* Type 2 samples:

WT_seq_plus: GCAGAGGACAAGGAGGATGATG

WT_seq_minus: CATCATCCTCCTTGTCCTCTGC

MUT_seq_plus: GAGGACAATTGTCGGAGGATGA

MUT_seq_minus: TCATCCTCCGACAATTGTCCTC

The genotype of a read-pair was called when the exact target sequences were found in both reads (no mismatches were allowed) and when both reads showed the same genotype. Overall, we received between 179729 and 248228 read-pairs per sample, and we were able to call a genotype in 92-96 % of these read-pairs. Finally, the total number of WT and MUT read-pairs was determined in each sample and the mutational burden was calculated as the percentage of read-pairs called mutant among all read-pairs for which a call could be made.

**RNA-Sequencing**

Megakaryocyte progenitors (MkPs; Lin^-^ c-kit^+^ sca-1^-^ CD41^+^ CD150^+^) from BM and SP of *Cdk6^+/+^* *CALR^+/+^*, *Cdk6^-/-^* *CALR^+/+^*, *Cdk6^+/+^* *CALR^del52^* and *Cdk6^-/-^* *CALR^del52^* mice were sorted into sterile round-bottom tubes and subsequently RNA isolation was performed using RNA-Solv® reagent and manufacturer protocol, followed by concentration determination using NanoDrop®.

Libraries for low-input bulk transcriptome sequencing were generated based on the SmartSeq3 protocol^7^ and sequenced on an Illumina NovaSeq Instrument. Raw sequencing reads were processed using Trimmomatic (version 0.39)^8^ to trim adapters and umi-tools (version 1.1.1)^9^ to remove unique molecular identifier sequences. Quality control of raw and trimmed reads was done with Fastqc (version 0.11.9).^10^ The processed reads were aligned against the primary assembly of the GRCm38 mouse reference genome (Gencode vM25)^11^ using STAR (version 2.7.6a).^12^ The gene model used was the Gencode vM25 primariy assembly annotation. Alignment QC was done using Qualimap (version 2.2.2a).^13^ Reads overlapping exons were then counted with the FeatureCounts program^14^ of the Subread package (version 2.0.1)^13^ and summarized as total counts per gene. Raw counts form all samples were combined into a single count table.

Differential gene expression analysis was performed in R (version 4.0.3)^15,16^ using the DESeq2 package.^17^ Sample genotype was coded as a factor with four levels based on *CALR* and *Cdk6* mutation status (Calr-mutant_Cdk6-wildtype, Calr-mutant_Cdk6-knockout, Calr-wildtype_Cdk6-knockout, and Calr-wildtype_Cdk6-wildtype (with the latter being set as the reference level. Genes with less than 50 counts in total (sum of the remaining 15 samples) were removed from the analysis. Differential gene expression analysis was performed using a model with genotype and sample date (batch) as fixed effects parameters.

The RNA sequencing data have been uploaded to ArrayExpress (Accession E-MTAB-15404).

***Gene Set Enrichment Analysis (GSEA)***

The analysis was done using the Broad Institute’s GSEA software version 4.2.1 or 4.1.0 (CD41 signatures) with the pre-ranked algorithm.^18,19^ Genes were ranked by -log10(P value) * sign(log2 fold change), where P value and log2 fold change were results from the differential gene expression analysis performed in MkPs. Genes were mapped to the human orthologs using the “Mouse_ENSEMBL_Gene_ID_Human_Orthologs_MSigDB.v7.2.chip” file (MSigDB) after removal of duplicate mappings.^20,21^ The applied scoring scheme was “classic”.

***Establishment of CD41-high and CD41-low gene expression signatures***

Gene expression signatures of CD41-high and CD41-low expressing cells were generated from published RNA seq data (Gene expression omnibus GSE132570). Differential gene expression analysis was performed in R (version 4.0.3)^15,16^ using the DESeq2 package^17^ comparing CD41-high and CD41-low expressing samples with and without the *JAK2^V614F^* mutation. The top 100 up- and down-regulated genes ranked by P value were regarded as marker genes for CD41-high and CD41-low expressing cells, respectively. These marker gene sets were used in gene set enrichment analysis against the results of the differential gene expression analysis performed in MkPs in-house.

Supplementary Table S1: Supplementary materials.

| RESOURCE/REAGENT | SOURCE | IDENTIFIER | Dilution used |
| --- | --- | --- | --- |
| Antibodies | | | |
| *Flow cytometry antibodies* | | | |
| *Mouse flow cytometry antibodies* | | | |
| TER-119-APC/Cy7 | Invitrogen | TER-119 | 1:200 |
| Gr-1-APC/Cy7 | Biozym | RB6-8C5 | 1:200 |
| CD11b-APC/Cy7 | Invitrogen | M1/70 | 1:200 |
| CD19-APC/Cy7 | eBioscience | eBio1D3 | 1:200 |
| CD3-APC/Cy7 | Biozym | 17A2 | 1:200 |
| Sca1-PE/Cy7 | eBioscience | D7 | 1:100 |
| CD86-PE/Cy7 | Biozym | GL-1 | 1:100 |
| c-Kit-PE-Cy5 | Invitrogen | 2B8 | 1:100 |
| CD150-BV510 | Biozym | TC15-12F12.2 | 1:100 |
| CD48-PE | Biozym | HM48-1 | 1:100 |
| CD41-APC | Biozym | MWReg30 | 1:100 |
| IFNAR1-PE | Biozym | MAR1-5A3 | 1:100 |
| CD45-BV605 | BD Biosciences | HM48-1 | 1:100 |
| CD45.1-PE | eBioscience | A20 | 1:100 |
| CD45.2-BV650 | Biozym | 104 | 1:100 |
| CD150-APC | Biozym | TC15-12F12.2 | 1:100 |
| CD41-FITC | eBioscience | eBioMWReg30 | 1:100 |
| CD150-Biotin | eBioscience | 9D1 | 1:100 |
| Streptavidin-BV650 | Biozym | Streptavidin | 1:1000 |
| CD45.2-APC/Cy7 | Biozym | 104 | 1:100 |
| Biotin Mouse Lineage Panel | BD Biosciences | 559971 | 1:200 |
| IgG-APC/Cy7 (H+L) | Invitrogen | Polyclonal | 1:500 |
| CDK6 | Invitrogen | PA5-27978 | 1:500 |
| AnnexinV-FITC | BD Biosciences | 556420 | 1:50 |
| FITC Anti-KI67 Set | BD Biosciences | 556026 | 1:10 |
| *Human flow cytometry antibodies* | | | |
| CD38-PE/Cy7 | Invitrogen | HIT2 | 1:200 |
| CD34-PE | Biozym | 581 | 1:100 |
| Cytokines | | | |
| *Mouse cytokines* | | | |
| SCF | VetMed (inhouse) |  | |
| IL-3 | R&D Systems | 403-ML | |
| IL-7 | R&D Systems | 407-ML | |
| IL-11 | ImmunoTools | 12340113 | |
| GM-CSF | R&D Systems | 415-ML-010 | |
| rmTPO | PeproTech | 314-15-100UG | |
| *Human cytokines* | | | |
| hSCF | PeproTech | AF300-07 | |
| hIL-3 | PeproTech | AF200-03 | |
| hIL-6 | PeproTech | AF200-06 | |
| hTPO | Miltenyi Biotec | 130-095-745 | |
| EPO | Janssen, Johnson & Johnson | ERYPO® FS | |
| Holo-transferrin | Sigma-Aldrich | T8158-100MG | |
| Insulin | Sigma-Aldrich | I2643-25MG | |
| Drugs and Inhibitors | | | |
| Palbociclib *(in vivo)* | Pfizer | PF-00080665 | |
| Palbociclib *(in vitro)* | Pfizer | PD-0332991 | |
| rmIFNα | MerckMillipore | IF009 | |
| Polyinosinic:polycytidylic acid (pIpC) | InvivoGen | Tlrl-pic-5 | |
| Ropeginterferon alfa-2b |  |  | |
| Chemicals, Peptides and Recombinant Proteins | | | |
| IMDM Medium | Sigma | I3390-500ML | |
| DMEM Medium | Sigma-Aldrich | D6429-500ML | |
| StemSPAN SFEM II Medium | StemCell Technologies | 9655 | |
| Fetal Bovine Serum (FBS) | Capricorn Scientific | FBS-12A | |
| PenStrep | Sigma | P4333-100ML | |
| L-Glutamine | Sigma-Aldirch | G7513-100ML | |
| TurboFect Transfection Reagent | Thermo Scientific | R0532 | |
| DNAse Type 1 | Sigma-Aldrich | DN25-1G | |
| 16% Formaldehyde Solution (w/v), methanol-free | Thermo Scientific | 28906 | |
| Tween 20 | ROTH | 9127.2 | |
| 2-Mercaptoethanol | Sigma | M3148-250ML | |
| DAPI | Sigma-Aldrich | D9542 | |
| AnnexinV Binding Buffer (10x) | Invitrogen | 00-0055-56 | |
| Mouse Methylcellulose Base Media | R&D Systems | HSC006 | |
| OSTEOSOFT^®^ | Sigma-Aldrich | 101728 | |
| Critical commercial assays | | | |
| MethoCult H4435 Enriched | StemCell Technolgies | 04445 | |
| Mouse Methylcellulose Base Media | R&D Systems | HSC006 | |
| BD Cytofix/Cytoperm Plus Fixation Permeabilization Kit | BD Biosciences | 555028 | |
| QIAamp DNA Micro Kit | Qiagen | 56304 | |
| QIAquick PCR Purification Kit | Qiagen | 28104 | |
| Deposited data | | | |
| GEO RNA-Seq in progress | hscs |  | |
| Experimental Models | | | |
| *Cell lines* | | | |
| Platinium-E (Plate-E) retroviral producer cell line | VetMed |  | |
| Murine HPC^LSK^ | Generated in-house | citation | |
| *Organisms/Strains* | | | |
| *Cdk6^-/-^* | VetMed | C57BL/6 | |
| VavCre-CALR^del52^ | Kindly provided by Anthony R. Green | C57BL/6 | |
| *Cdk6^-/-^* VavCre-CALR^del52^ | VetMed | C57BL/6 | |
| NSG | Charles River | NOD.Cg-Prkdcscid Il2rgtm1Wjl/SzJ | |
| Recombinant DNA | | | |
| CALR^del52^-GFP in pMSCV | Cloned in-house |  | |
| Software and Algorithms | | | |
| GraphPad Prism (8.4.3.686) | GraphPad Software |  | |
| Cytexpert (2.4.0.28) | Beckman Coulter |  | |
| Venny software 2.1. | By Juan Carlos Oliveros, BioinfoGP, CNB-CSIC |  | |
| R 4.2.2 | R foundation | https://www.r-project.org/ | |
| RStudio 2024.09.1 | Rstudio | https://posit.co/download/rstudi o-desktop/ | |
| GSEA 4.1.0 or 4.2.1 | Broad Institute | https://www.gsea-msigdb.org/gsea/ | |

Supplementary Table S2: Information on primary patient samples.

**ASS = Acetylsalicylic acid*

| Patient | Disease | Mutation | Tissue | Status/Therapy |
| --- | --- | --- | --- | --- |
| 1 | ET (follow up) | *JAK2^V617F^* | BM | Follow-up (only ASS*). *JAK2* burden = 34.9% |
| 2 | PMF (prefibrotic) | *JAK2^V617F^* | BM | Initial diagnosis. *JAK2* burden = 22.3% |
| 3 | ET (follow up) | *JAK2^V617F^* | BM | Follow-up (only ASS*). *JAK2* burden = 7.8% |
| 4 | ET | *CALR* Type 1 | BM | Initial diagnosis |
| 5 | PMF | *CALR* Type 1 | BM | Initial diagnosis |
| 6 | ET | *CALR* Type 1 | BM | Initial diagnosis |
| 7 | PMF | *CALR* Type 1 | BM | Initial diagnosis |
| 8 | NHL | n.a. | BM | Initial diagnosis |
| 9 | NHL | n.a. | BM | Initial diagnosis |
| 10 | Lymphoma | n.a. | BM | Initial diagnosis |

Supplementary Table S3: Information on primary patient samples used for CFA with VAF analysis.

| Patient | Disease | Mutation | Tissue | Status/Therapy |
| --- | --- | --- | --- | --- |
| 11 | Lymphoma (w/o infiltration) | n.a. | BM | Initial diagnosis |
| 12 | NHL (w/o infiltration) | n.a. | BM | Initial diagnosis |
| 13 | NHL (w/o infiltration) | n.a. | BM | Follow-up (Remission) |
| 14 | ET | *JAK2^V617F^* | BM | Follow-up (only ASS*). JAK2 burden = 34.9% |
| 15 | ET | *JAK2^V617F^* | BM | Follow-up (only ASS*). JAK2 burden in PB = 7.8% |
| 16 | PV | *JAK2^V617F^* | BM | Follow-up (Phlebotomy). JAK2 burden = 23.5% |
| 17 | ET | *CALR* Type 1 | BM | Initial diagnosis |
| 18 | PMF | *CALR* Type 1 | BM | Initial diagnosis |
| 19 | ET | *CALR* Type 2 | BM | Initial diagnosis |
| 20 | PMF | *CALR* Type 2 | BM | Follow-up, No Cytoreductive therapy (only supportive) |

**ASS = Acetylsalicylic acid*


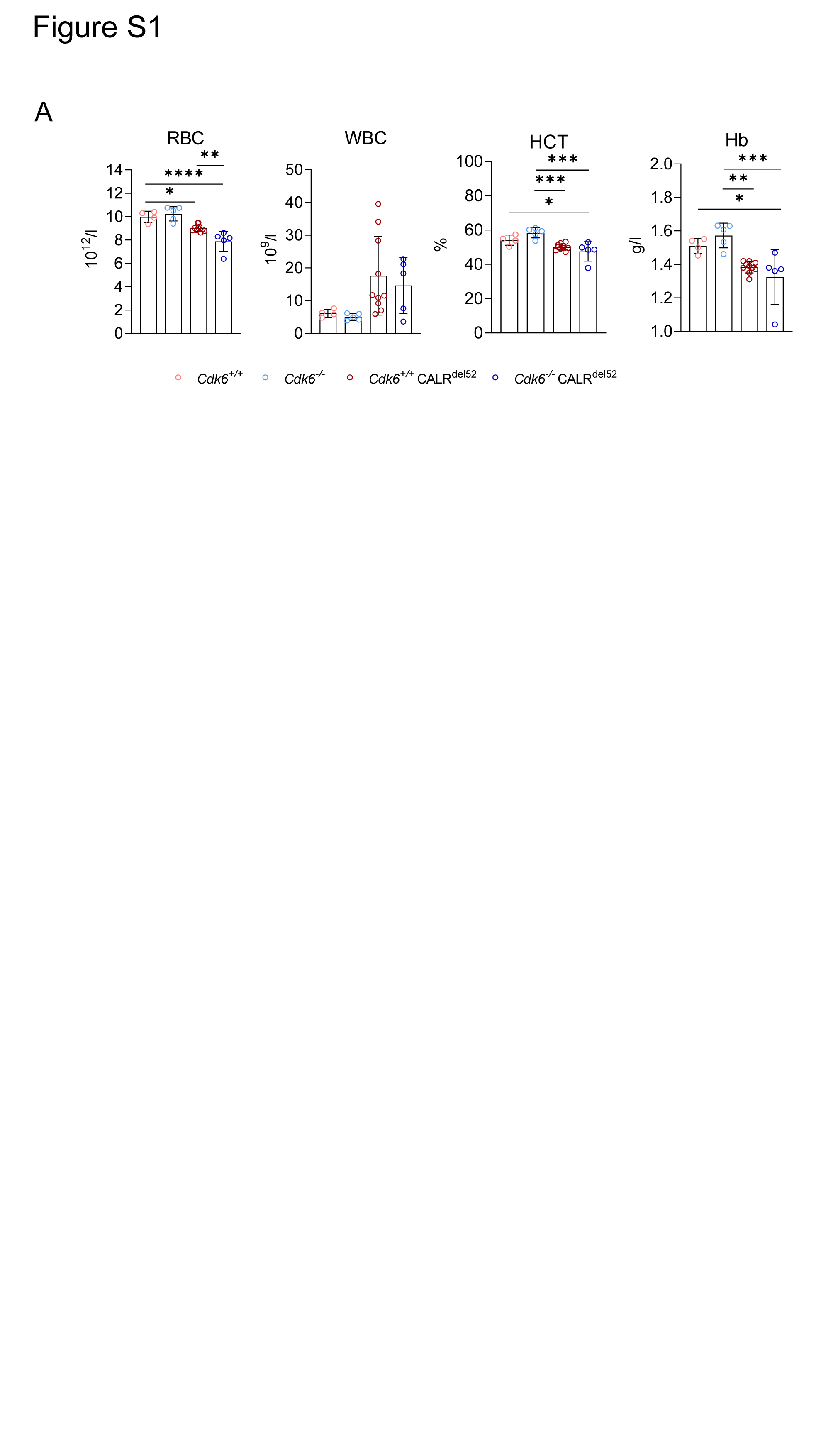


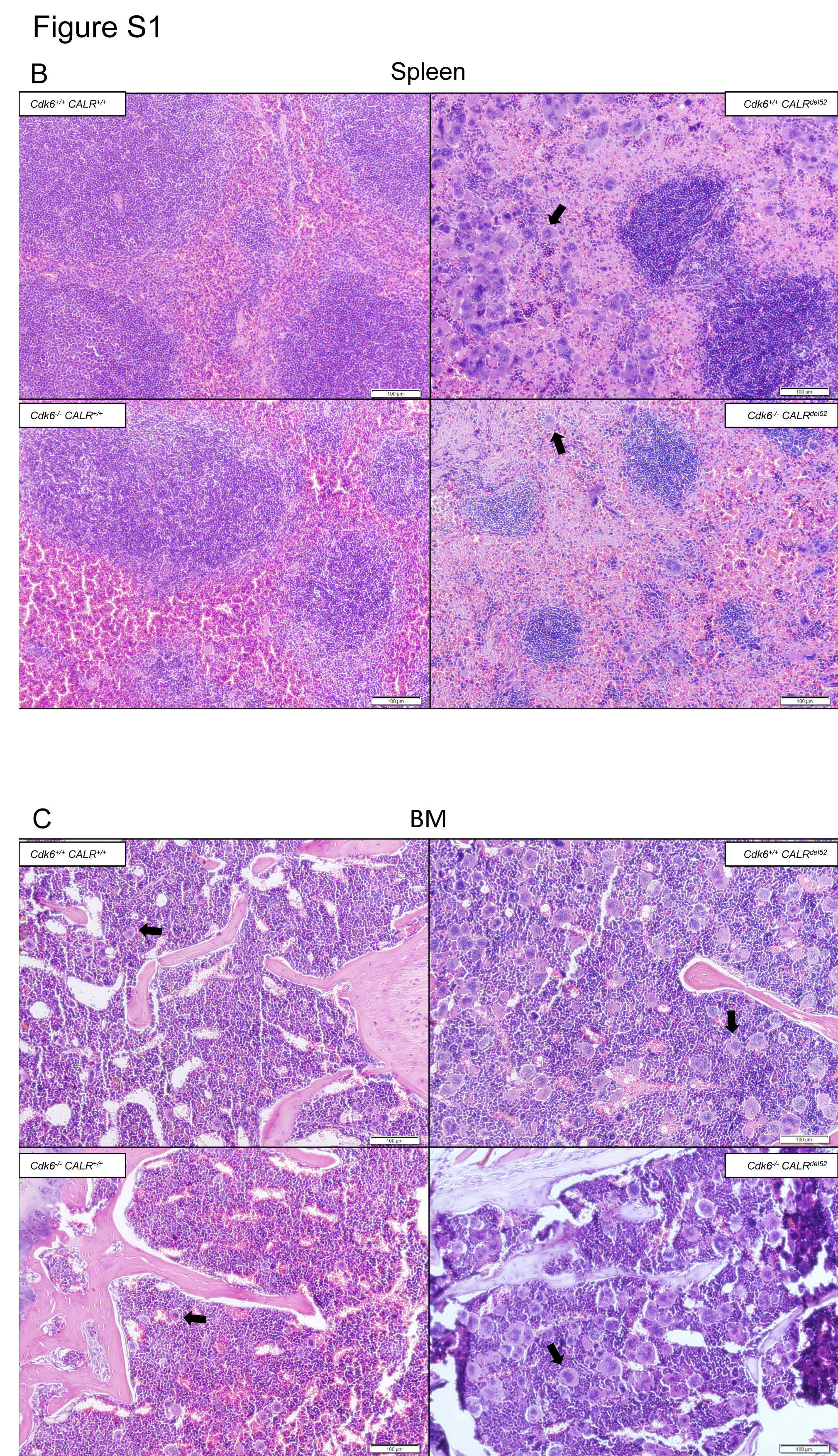


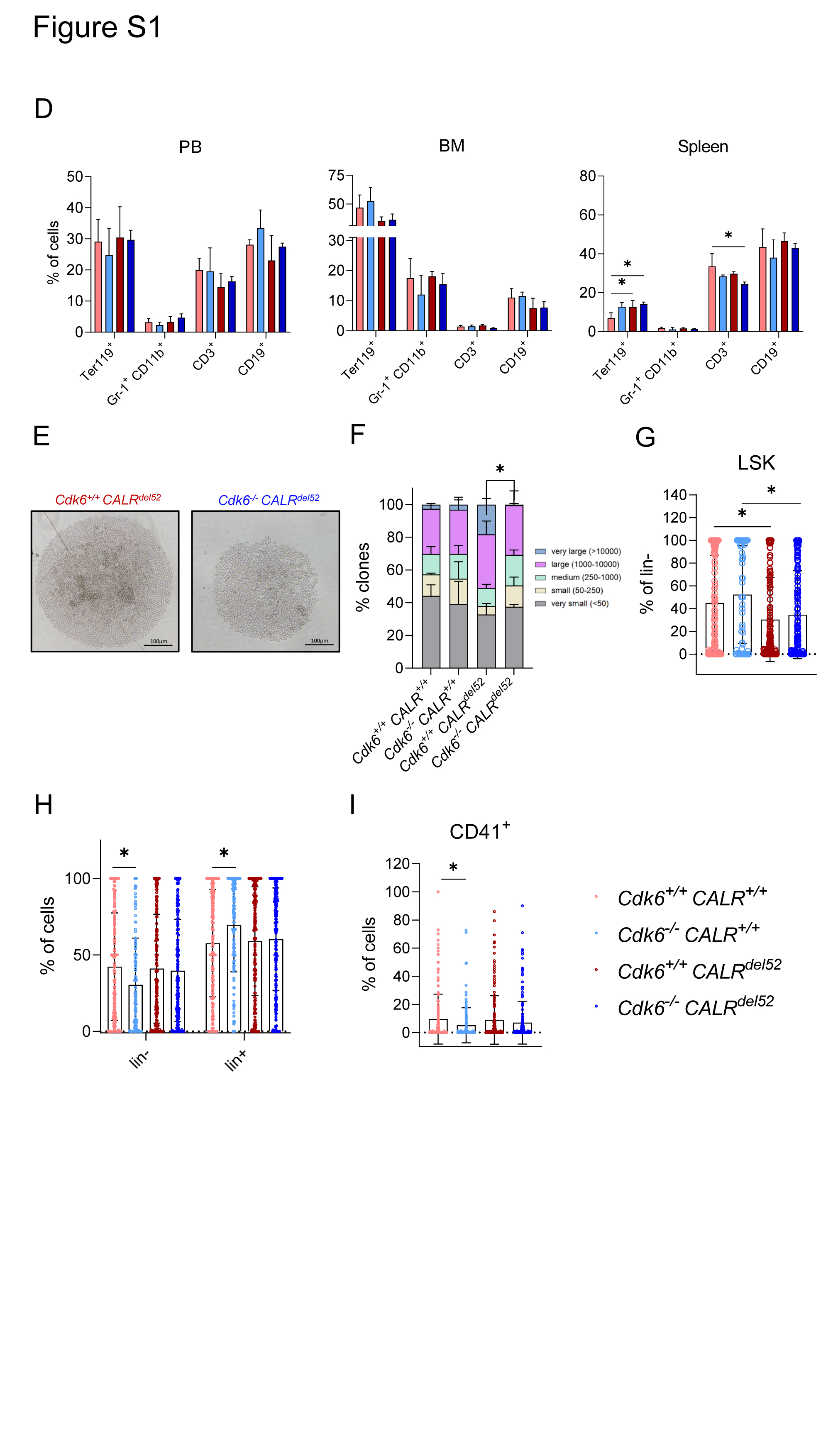


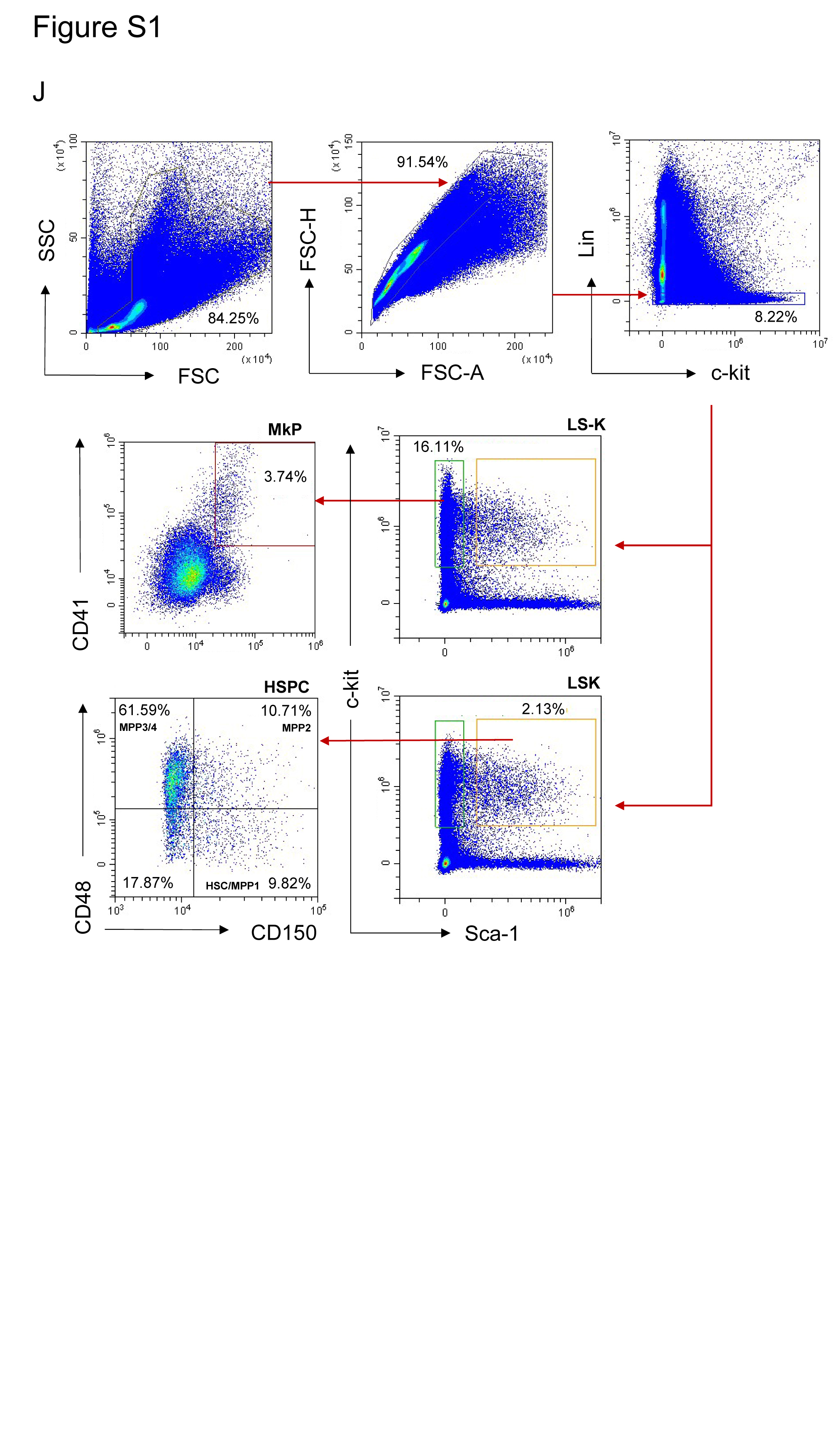


**Supplementary Figure S1: *Cdk6* loss reduces the MPN phenotype in *CALR*-mutant mice in a cell intrinsic manner**

(**a**) Red blood cell (RBC) in 10^12^/l, white blood cell (WBC) 10^9^/l, hematocrit (HCT) in % and hemoglobin (Hb) in g/l of 8-week-old *Cdk6^+/+^ CALR^+/+^* (light red, n=6), *Cdk6^-/-^* *CALR^+/+^* (light blue, n=8), *Cdk6^+/+^* *CALR^del52^* (red, n=13) and *Cdk6^-/-^* *CALR^del52^* (blue, n=6) mice obtained via veterinary animal blood counter (vetABC).

(**b**) Representative pictures of H&E staining of Spleen and (**c**) BM (10x magnification; 100µm) from 8-week-old *Cdk6^+/+^ CALR^+/+^*, *Cdk6^-/-^* *CALR^+/+^*, *Cdk6^+/+^* *CALR^del52^* and *Cdk6^-/-^* *CALR^del52^* mice (n≥2). Megakaryocyte cells are highlighted with black arrows.

(**d**) Flow cytometric analysis of erythroid (Ter119^+^), myeloid (Gr-1^+^ CD11b^+^) and lymphoid (CD3^+^, CD19^+^) lineages in peripheral blood (PB, left), bone marrow (BM, middle) and spleen (SP, right) of 8-week-old *Cdk6^+/+^ CALR^+/+^*, *Cdk6^-/-^* *CALR^+/+^*, *Cdk6^+/+^* *CALR^del52^* and *Cdk6^-/-^* *CALR^del52^* mice. Blood n≥4, BM n≥4, SP n≥2. Error bars represent mean±SD. **P* <0.05; ***P* <0.01; ****P* < 0.001; *****P* < 0.0001 by ordinary one-way ANOVA followed by Tukey’s multiple comparison test.

(**e**) Representative picture of sorted HSC/MPP1 cells from *Cdk6^+/+^* *CALR^del52^* (red) and *Cdk6^-/-^* *CALR^del52^* (blue) mice *in vitro*.

(**f**) Colony size categorization by cell numbers obtained via flow cytometry of single cell-cultured HSC/MPP1 cells from 8-week-old *Cdk6^+/+^ CALR^+/+^*, *Cdk6^-/-^* *CALR^+/+^*, *Cdk6^+/+^* *CALR^del52^* and *Cdk6^-/-^* *CALR^del52^* mice. Clone sizes were categorized into very small (<50), small (50-250), medium (250-1000), large (1000-10000) and very large (>10000) cell numbers and percentages were calculated.

(**g**) Cell percentages of LSKs, (**h**) lineage (lin) negative/positive cells and (**i**) CD41^+^ cells obtained from single cell HSC/MPP1 *in vitro* cultures of *Cdk6^+/+^ CALR^+/+^* (light red), *Cdk6^-/-^* *CALR^+/+^* (light blue), *Cdk6^+/+^* *CALR^del52^* (red) and *Cdk6^-/-^* *CALR^del52^* (blue) mice after 7 days of culture. 192 wells (each well representing a clone) per genotype (2 mice each) were analyzed and cell percentages derived from each clone were plotted (number of cells per clone). Error bars represent mean±SD. **P* <0.05; ***P* <0.01; ****P* < 0.001; *****P* < 0.0001 by ordinary one-way ANOVA followed by Tukey’s multiple comparison test.

(**j**) Gating strategy for Lin-, MkP (megakaryocyte progenitor, Lin^-^ Sca-1^+^ c-kit^+^ CD41^+^ CD150^+^), LSK (Lin^-^ Sca-1^+^ c-kit^+^) and HSPC: HSC/MPP1 (Lin^-^ Sca-1^+^ c-kit^+^ CD150^+^ CD48^-^), MPP2 (Lin^-^ Sca-1^+^ c-kit^+^ CD150^+^ CD48^+^) and MPP3/4 (Lin^-^ Sca-1^+^ c-kit^+^ CD150^-^ CD48^+^) cell populations.


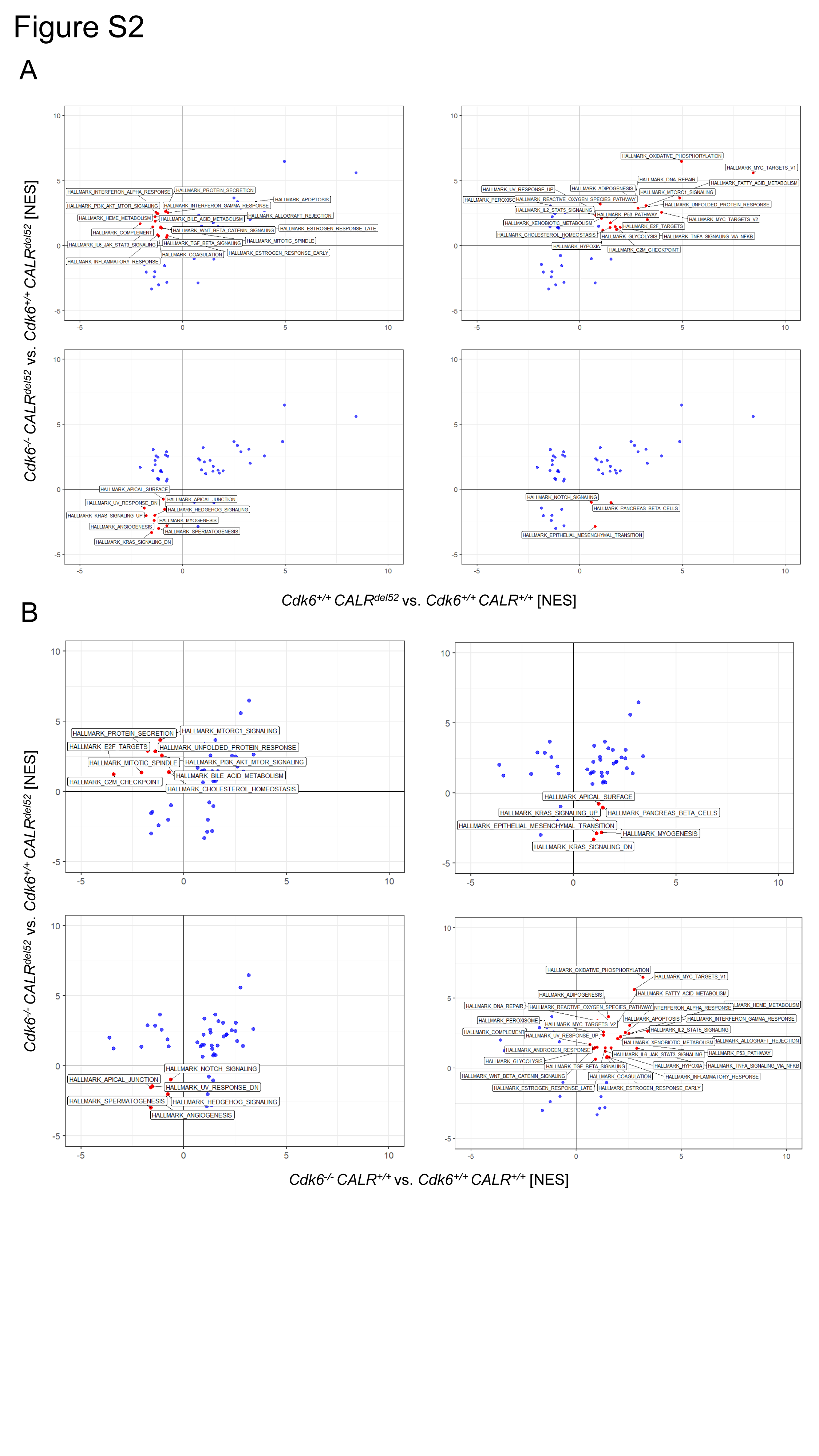


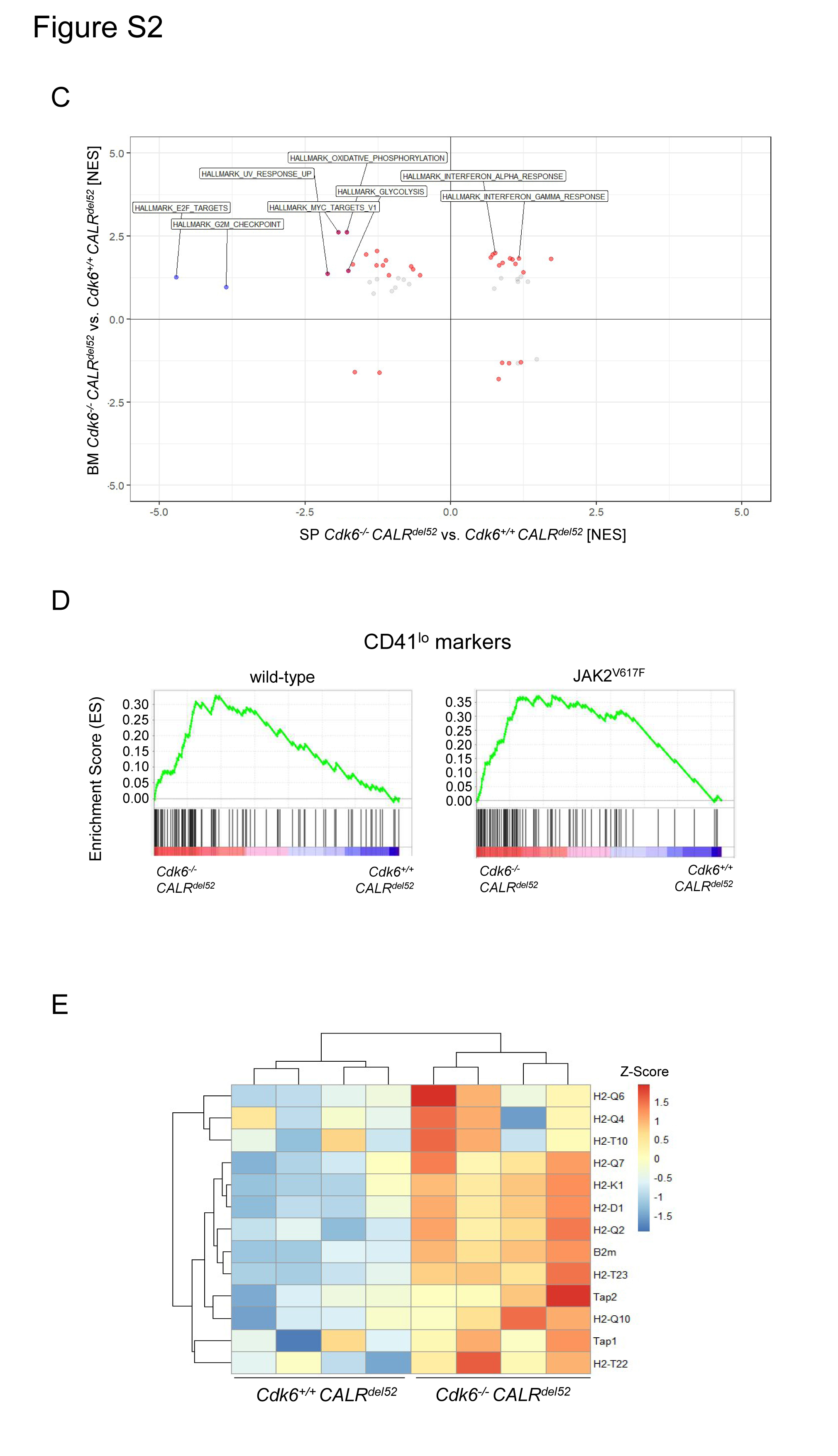


**Supplementary Figure S2: *Cdk6* knockout MkPs transcriptionally upregulate IFN-associated pathways**

(**a**) Scatterplot of *Cdk6^-/-^* *CALR^del52^* vs *Cdk6^+/+^* *CALR^del52^* (y-axis) and *Cdk6^+/+^* *CALR^del52^* vs *Cdk6^+/+^ CALR^+/+^* (x-axis) and (**b**) *Cdk6^-/-^* *CALR^del52^* vs *Cdk6^+/+^* *CALR^del52^* (y-axis) and *Cdk6^-/-^* *CALR^+/+^* vs *Cdk6^+/+^ CALR^+/+^* (x-axis) datasets showing common and distinct (opposite) regulated pathway enrichment.

(**c**) Scatterplot of *Cdk6^-/-^* *CALR^del52^* vs *Cdk6^+/+^* *CALR^del52^* BM MkP’s (y-axis) and *Cdk6^-/-^* *CALR^del52^* vs *Cdk6^+/+^* *CALR^del52^* SP MkP’s (x-axis) datasets showing common and distinct (opposite) regulated pathway enrichment. Significantly enriched pathways (FDR < 0.1) from both BM and SP datasets (dark red), BM only (red) and SP only (blue) are shown. Pathways not significant (FDR > 0.1) are marked in grey.

(**d**) Enrichment plot from gene set enrichment analysis (GSEA) using HALLMARK-database from the *Cdk6^-/-^* *CALR^del52^* vs *Cdk6^+/+^* *CALR^del52^* MkP dataset using a publicly available CD41-lo gene signature from wild-type (left) and *JAK2^V617F^* (right) cells.

(**e**) Heatmap displaying genes significantly deregulated within the *Cdk6^-/-^* *CALR^del52^* vs *Cdk6^+/+^* *CALR^del52^* MkP dataset using publicly available MHCII^hi^/MHCII^lo^ gene signature.


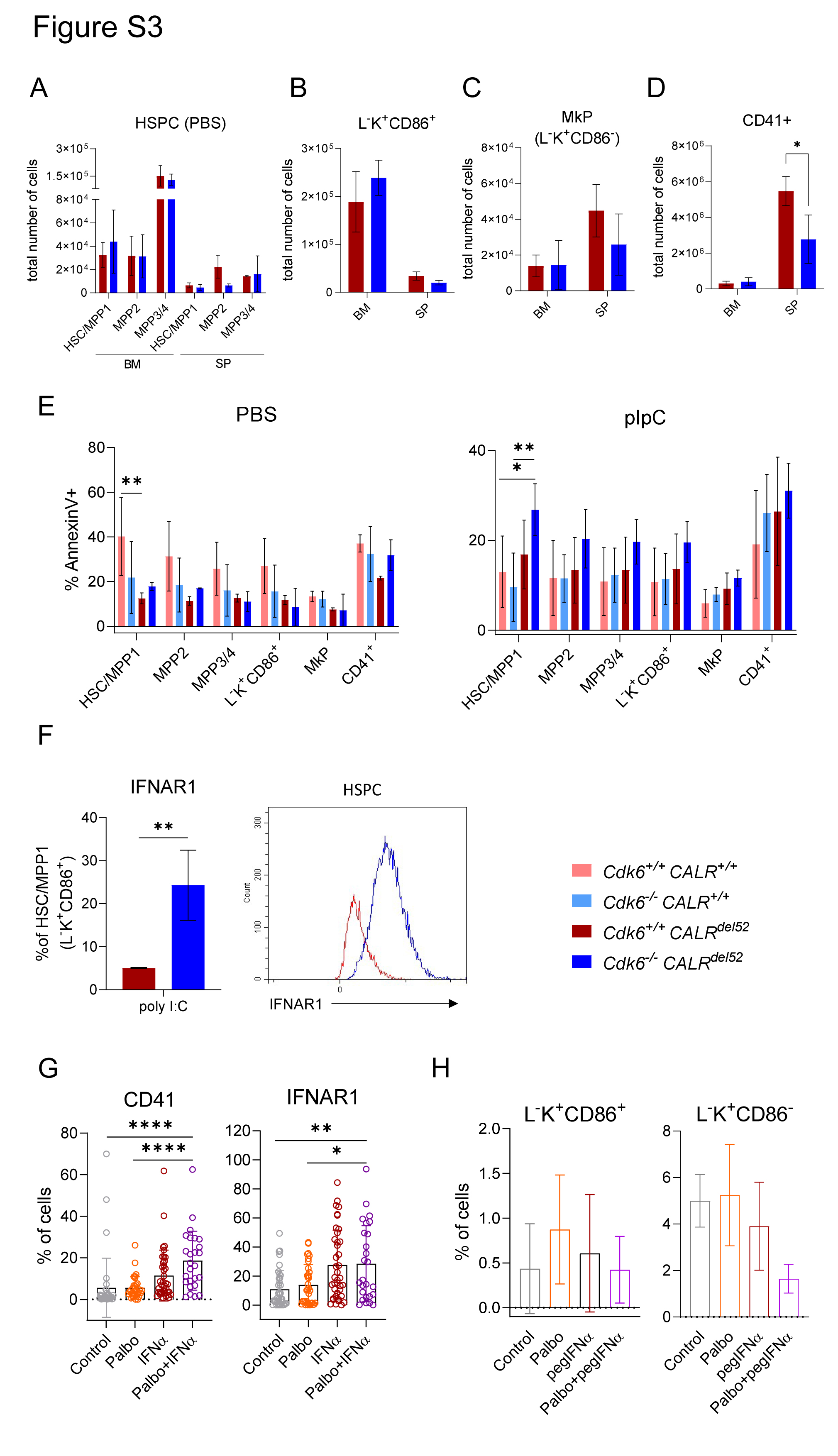

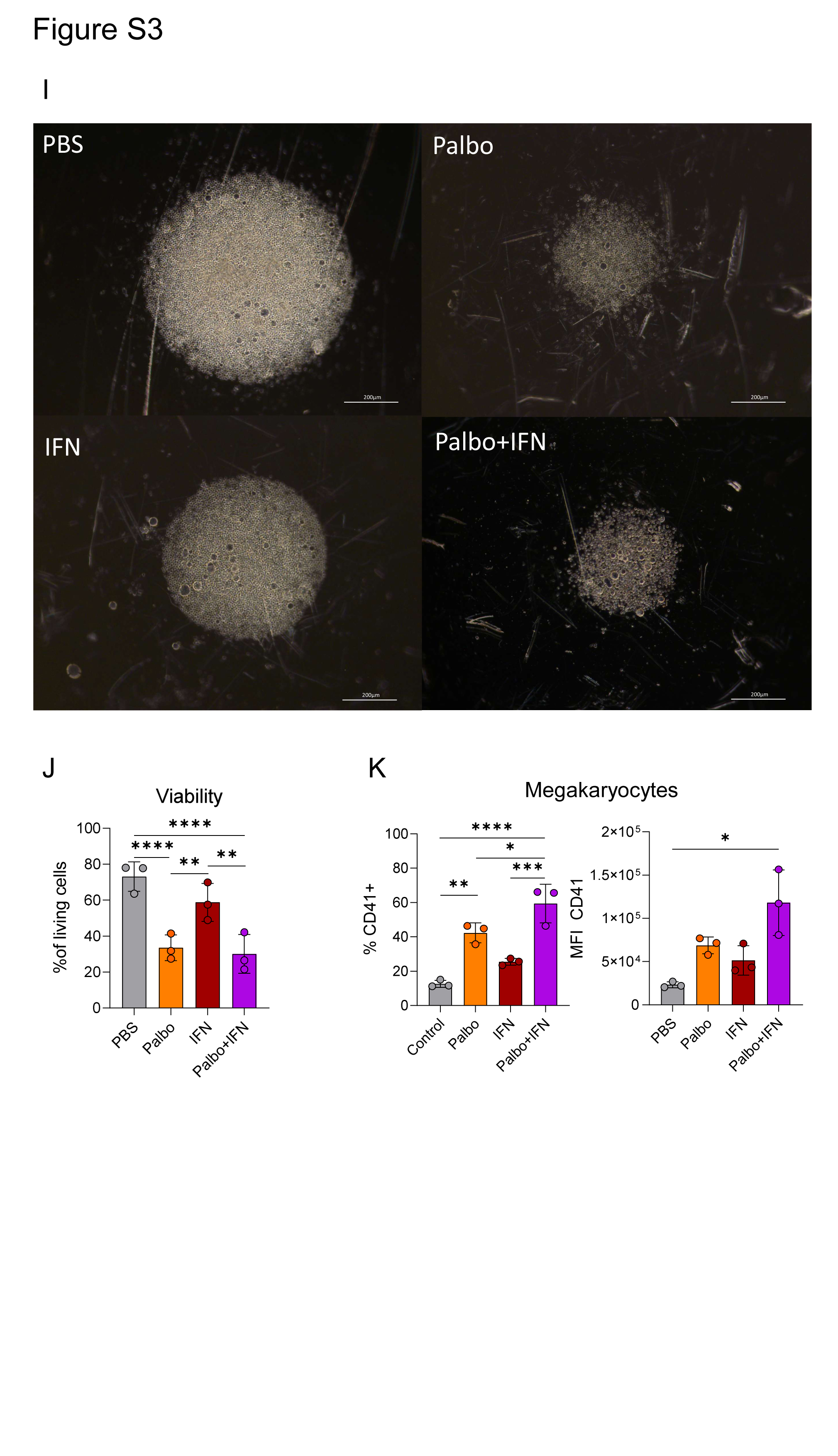


**Supplementary Figure S3: CDK6 ablation increases IFN**α**-induced apoptosis and IFNAR1 expression in *CALR^del52^* HSPCs**

(**a**) Flow cytometric analysis showing total cell numbers of HSPCs within BM and SP treated with PBS only, (**b**) L^-^K^+^CD86^+^, (**c**) MkP (from L^-^K^+^CD86^+^) and (**d**) CD41^+^ cells within BM and SP of pIpC-treated *Cdk6^+/+^* *CALR^del52^* (red) and *Cdk6^-/-^* *CALR^del52^* (blue) mice. Error bars represent mean±SD. n≥2. **P* <0.05 by unpaired two-tailed student’s t-test.

(**e**) Annexin-V/DAPI flow cytometry staining showing percentages of HSC/MPP1, MPP2, MPP3/4, L^-^K^+^CD86^+^, MkP and CD41^+^ cells in the BM of PBS (left) and pIpC-treated (right) *Cdk6^+/+^* *CALR^del52^* and *Cdk6^-/-^* *CALR^del52^* mice. n≥2. **P* <0.05; ***P* <0.01 by ordinary one-way ANOVA followed by Tukey’s multiple comparison test.

(**f**) Expression of IFNAR1 in HSC/MPP1 populations of pIpC-treated *Cdk6^+/+^* *CALR^del52^* (red) and *Cdk6^-/-^* *CALR^del52^* (blue) mice measured by flow cytometry (left). Histogram overlay between *Cdk6^+/+^* *CALR^del52^* (red) and *Cdk6^-/-^* *CALR^del52^* (blue) HSPC populations expressing IFNAR1 (right). Error bars represent mean±SD. n≥2. ***P* <0.01 by unpaired two-tailed student’s t-test.

(**g**) Flow cytometric analysis of CD41 (left) and IFNAR1 (right) expression from HSC/MPP1 single cell-sorted clones of *Cdk6^+/+^* *CALR^del52^* mouse BM after 7 days of culturing in StemSPAN SFEM II HSC expansion media with hIL-11 and SCF, treated with PBS control (grey), 200nM palbociclib (orange), 100U IFNα (red) or a 200nM palbociclib/100U IFNα combination (purple). 48 wells (each well representing a clone) per treatment condition were analyzed. Data are presented with mean±SD of two independent experiments and n=2 mice per experiment (4 total). ****P* < 0.001; *****P* < 0.0001 by ordinary one-way ANOVA followed by Tukey’s multiple comparison test.

(**h**) Colony formation assay using fully supplemented murine methylcellulose with 100 bulk-sorted LSK cells from *Cdk6^+/+^* *CALR^del52^* mouse BM treated with PBS control (grey), 200nM palbociclib (orange), 600ng pegIFNα (red) or a 200nM palbociclib/600ng pegIFNα combination (purple). Flow cytometric analysis was carried out 7 days post-cultivation and analyzed for percentages of L^-^K^+^CD86^+^ (left) and L^-^K^+^CD86^-^ (right) cells.

(**i**) Representative picture of megakaryocyte (MK) assay using 100 bulk-sorted LSK cells from *Cdk6^+/+^* *CALR^del52^* mouse BM treated with PBS control, 200nM Palbociclib, 100U rmIFNα and a 200nM palbociclib/100U rmIFNα combination (4x magnification; 200µm). (**j**) Cell viability and (**k**) quantification of differentiated CD41^+^ cells were determined by flow cytometry. Error bars represent mean±SD. n=3 per condition. **P* <0.05; ***P* <0.01; ****P* <0.001; *****P* <0.0001 by ordinary one-way ANOVA followed by Tukey’s multiple comparison test.

**
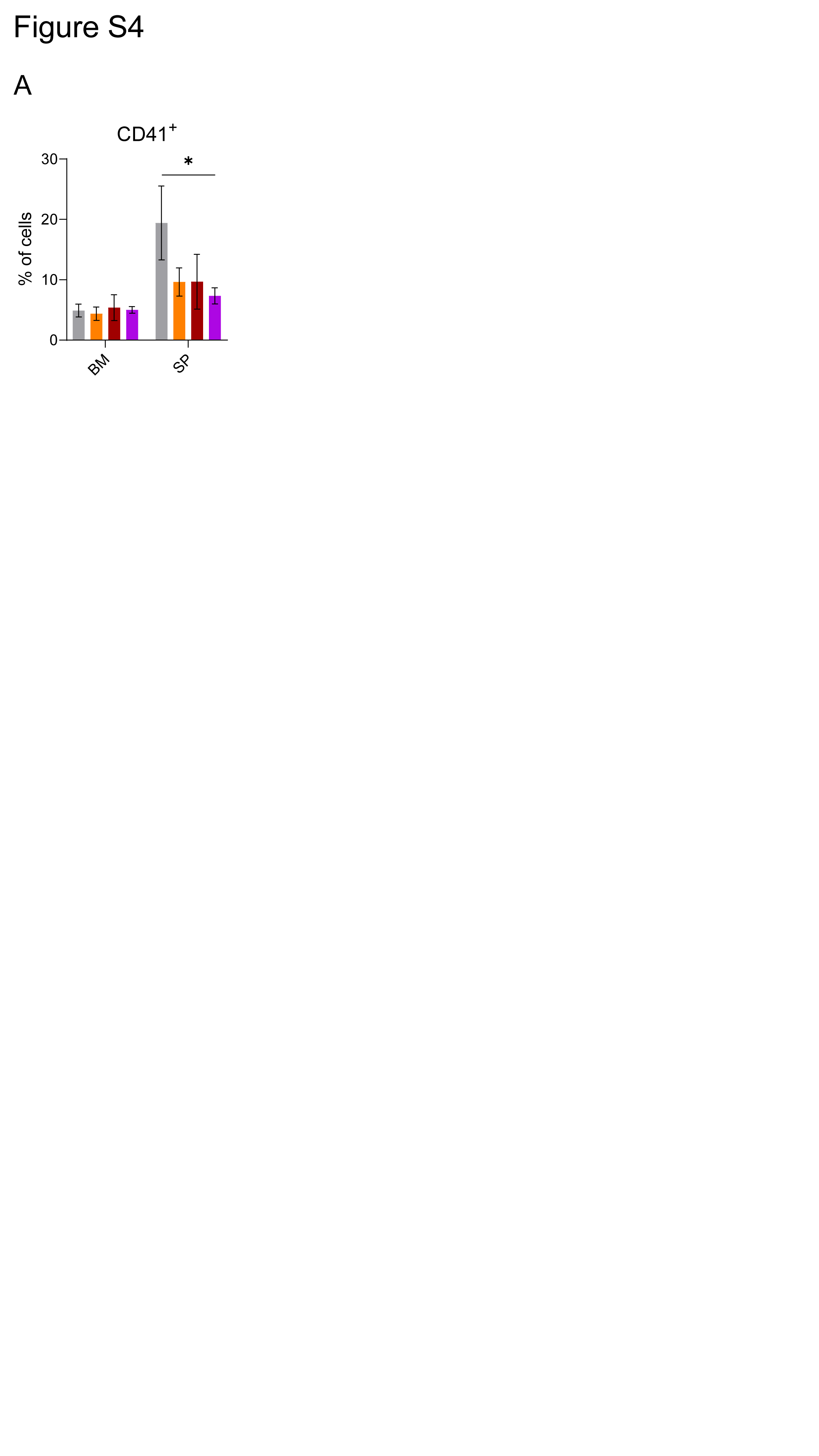
**

**Supplementary Figure S4: Concomitant CDK4/6 kinase inhibition and pegIFNα treatment reduce the *CALR*-mutant disease phenotype**

**(a)** Flow cytometric analysis of CD41^+^ cells in BM and SP of NSG recipients.

Error bars represent mean±SD. n=3 per condition. **P* <0.05 by two-way ANOVA followed by Tukey’s multiple comparison test.

**
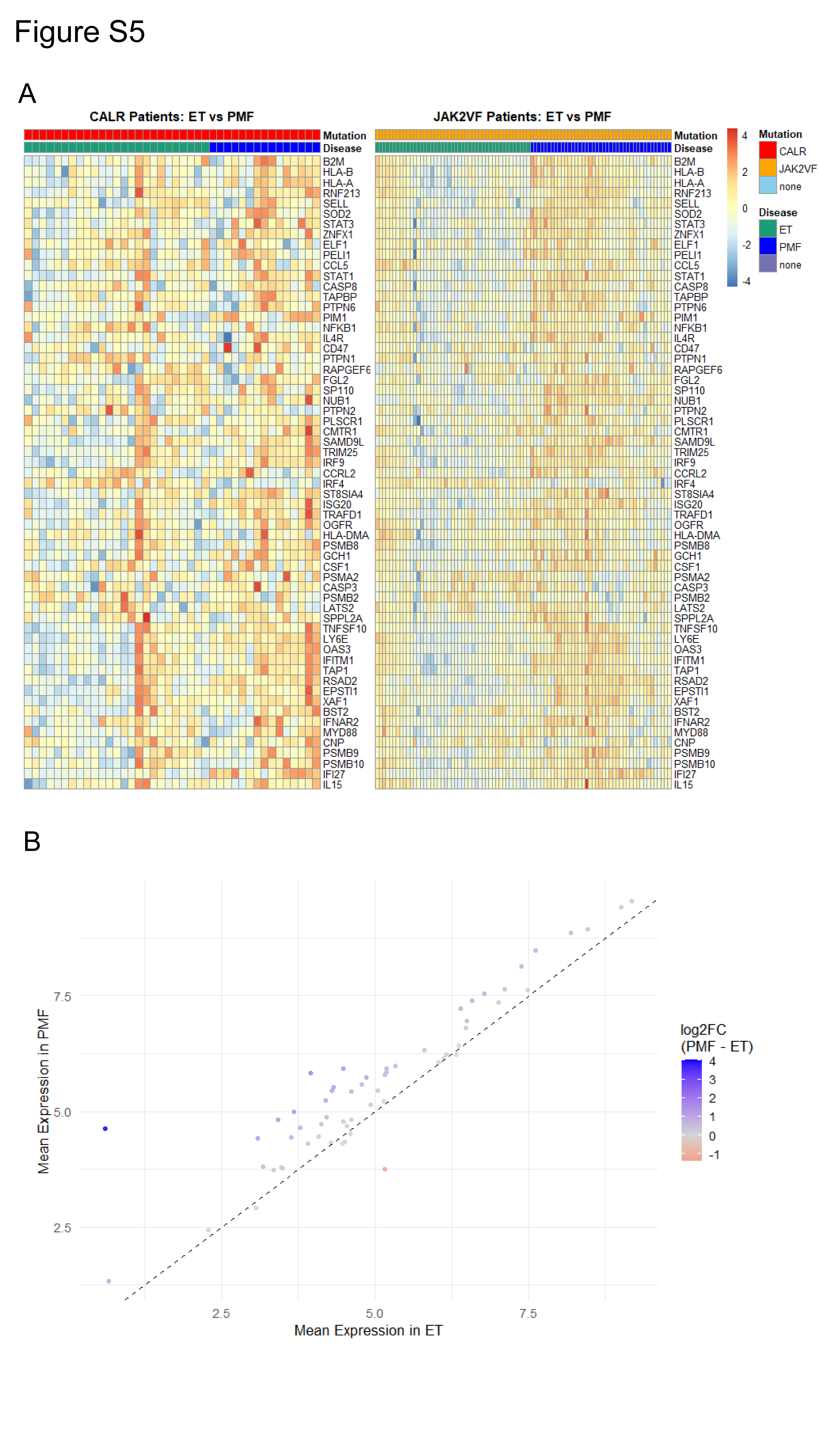
**


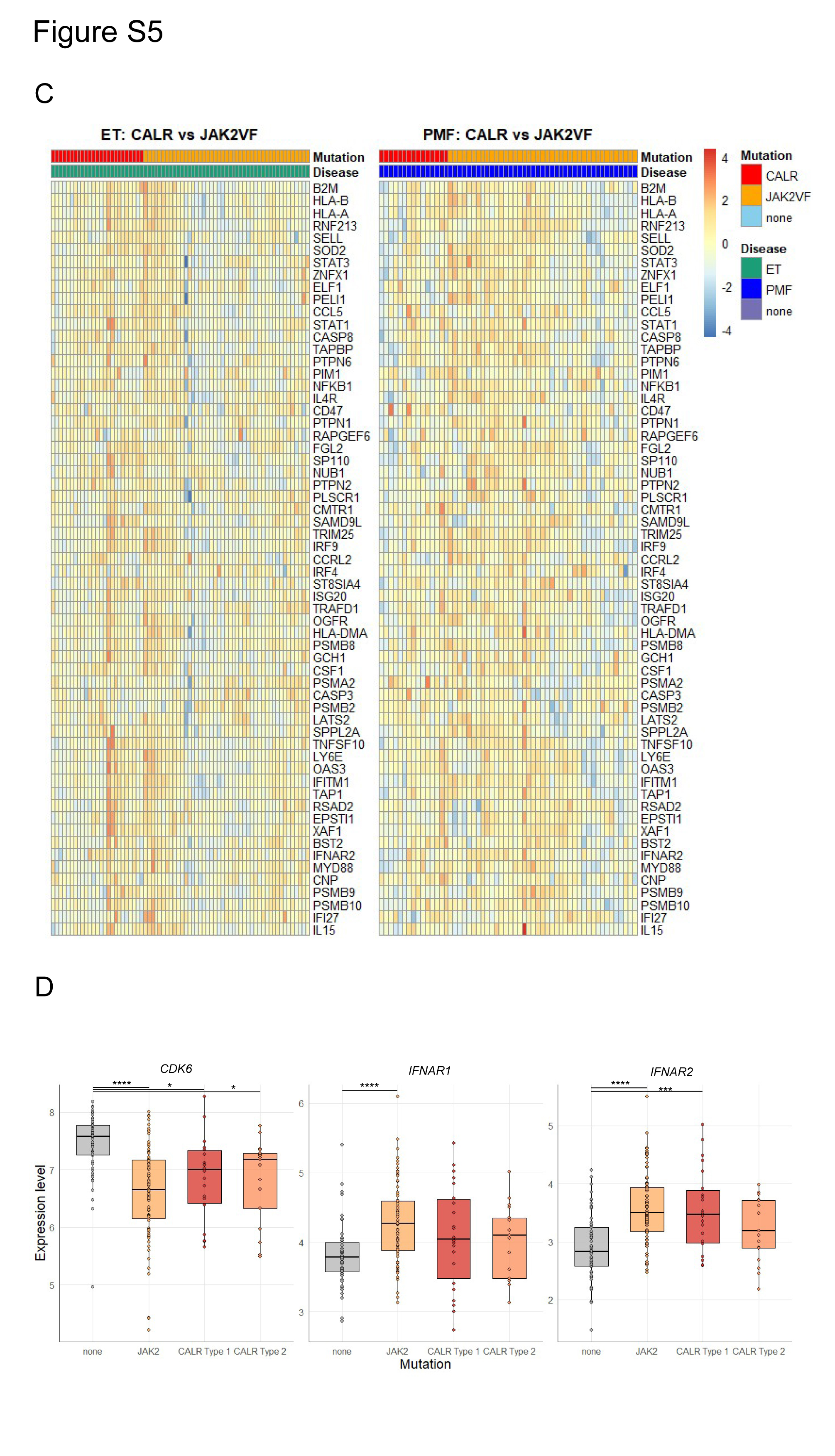


**Supplementary Figure S5: Interferon alpha and gamma gene signatures are preferentially upregulated in PMF patients independent of mutation**

**(a)** Heatmaps showing RNA-Seq data of BM and PB samples from a 230-patient cohort comprising 64 controls without mutation or disease, 86 *JAK2^V617F^* mutations, 39 *CALR* mutations. The heatmap was generated using the pheatmap package in R-studio. Plotted are log2CPM normalized counts that have been scaled across samples. Clustering was done using the ward.D2 clustering method. RNA-Seq gene signature of Interferon-alpha and Interferon-gamma signaling pathways obtained from GSEA of *Cdk6^-/-^* *CALR^del52^* vs *Cdk6^+/+^* *CALR^del52^* MkPs were used to determine this signature in ET and PMF MPN patients harboring a *CALR* mutation (left) or *JAK2^V617F^* mutation (right).

**(b)** Linear regression analysis of RNA-Seq gene signature of Interferon-alpha and Interferon-gamma signaling pathways obtained from GSEA of *Cdk6^-/-^* *CALR^del52^* vs *Cdk6^+/+^* *CALR^del52^* MkPs in ET versus PMF patients.

(**c**) Heatmap showing the comparison of Interferon-alpha and Interferon-gamma gene signatures in ET patients (left) or PMF patients (right) harboring either a *CALR* or *JAK2^V617F^* mutation.

(**d**) Bar plots of 22 *CALR* Type 1, 17 *CALR* Type 2, 86 *JAK2^V617F^* and 64 control patient samples from BM and PB, clustered according to their mutation and irrespective of disease, showing *CDK6*, *IFNAR1* and *IFNAR2* expression levels. The plots were generated using the ggplot2 package in R-studio. **P* <0.05; ***P* <0.01; ****P* <0.001; *****P* <0.0001 by ordinary one-way ANOVA followed by Tukey’s multiple comparison test.

**
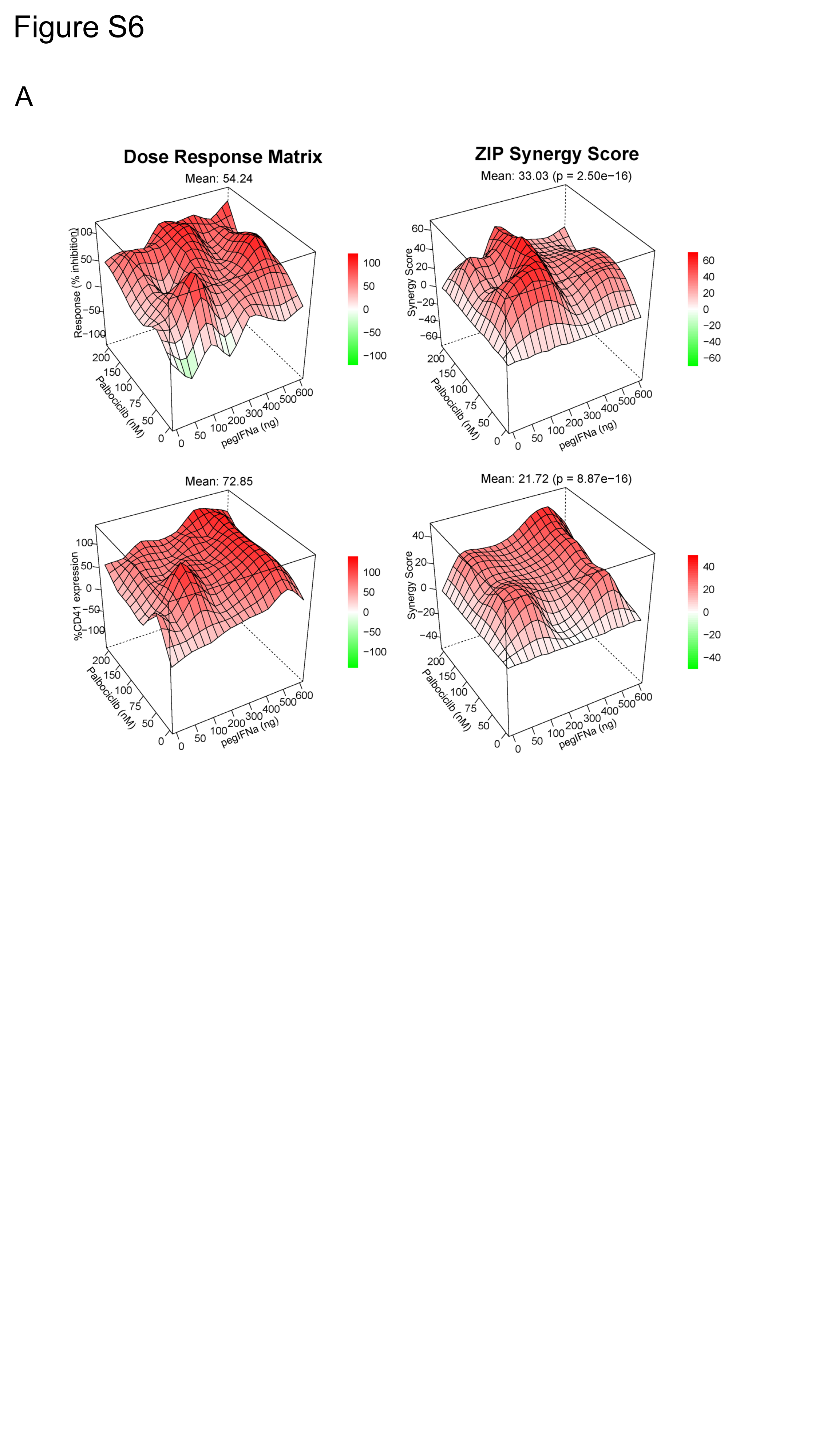
**
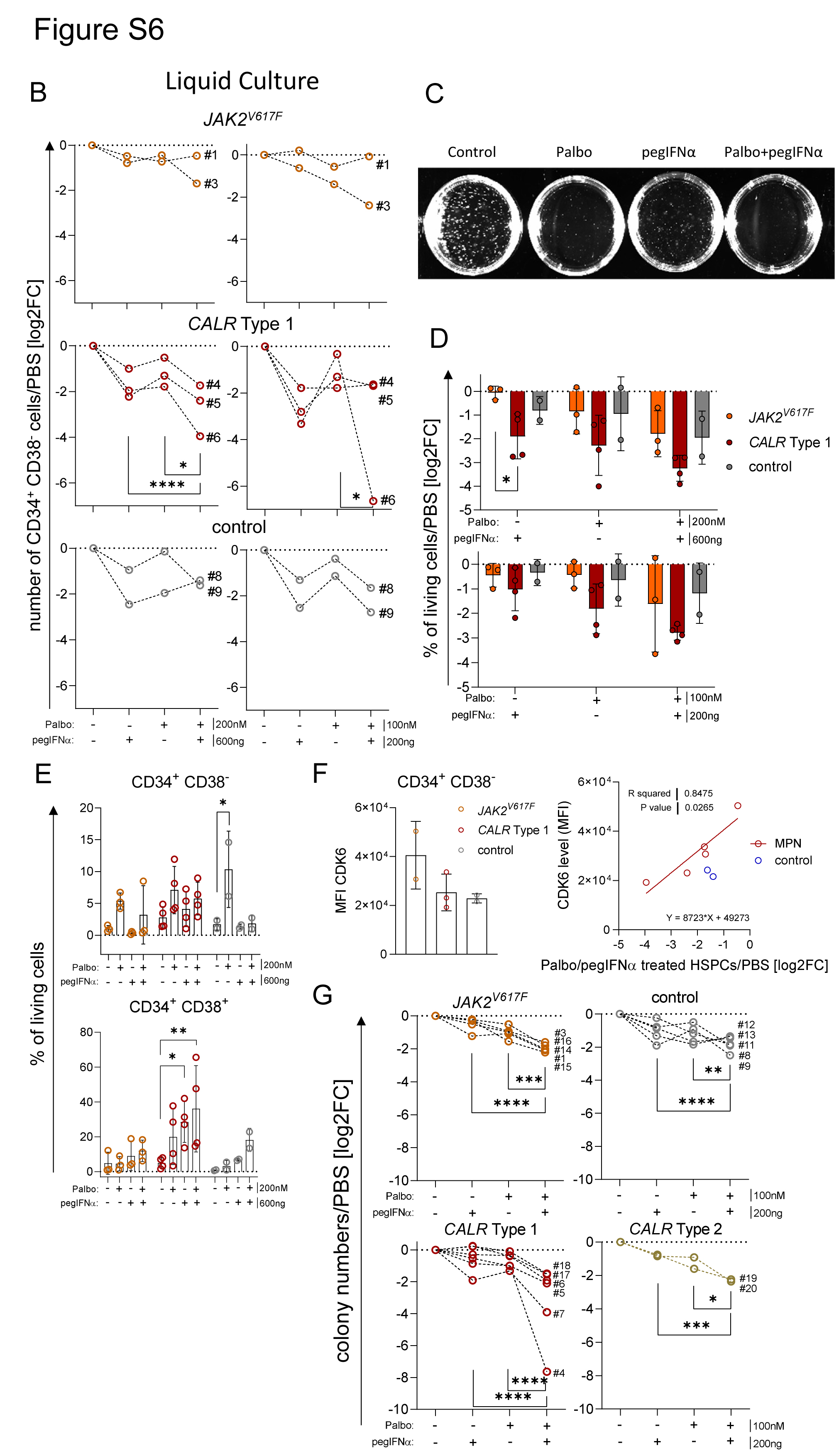


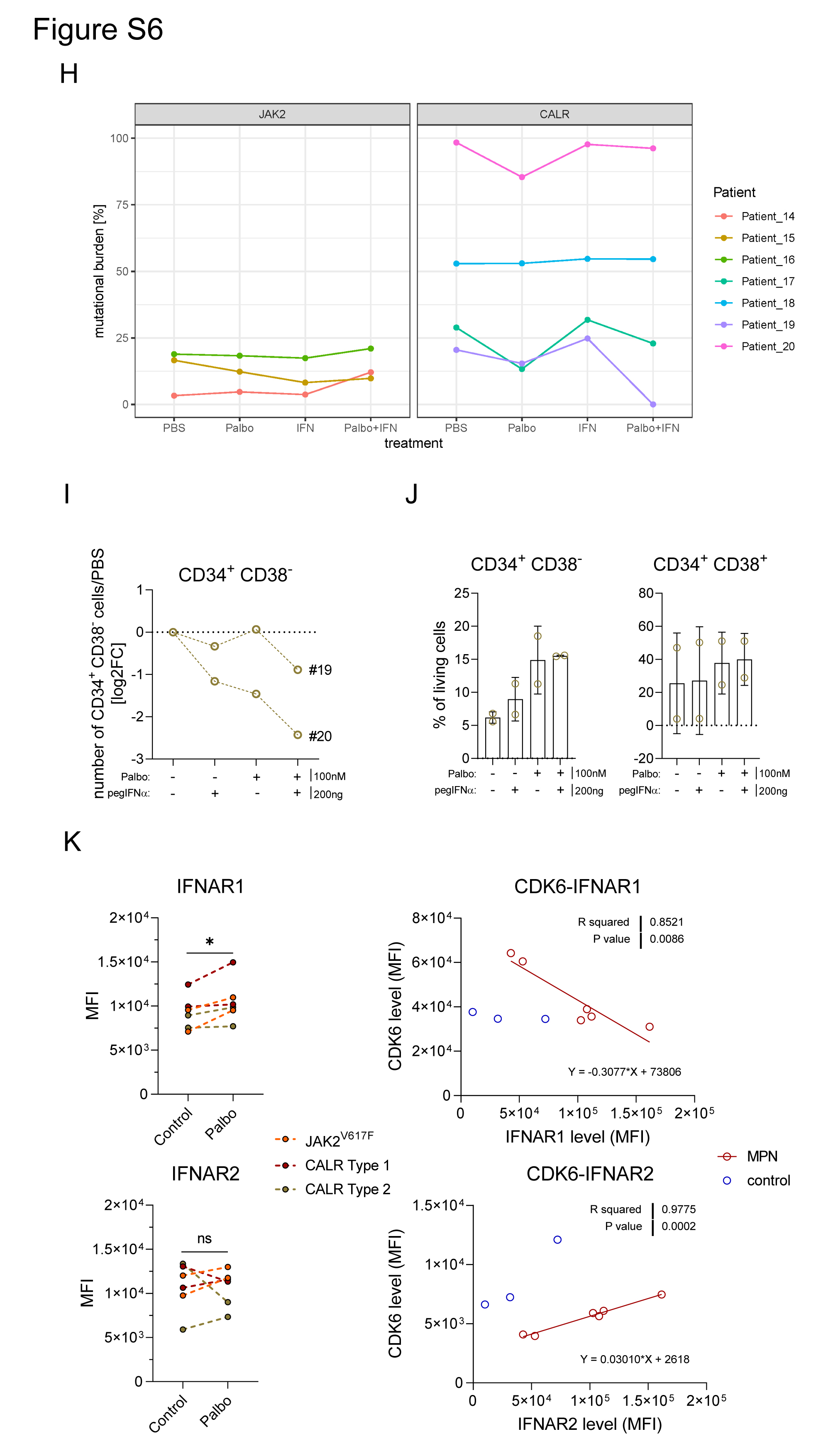


**Supplementary Figure S6: CDK4/6 inhibitor palbociclib and pegIFNα act synergistically and allow reduction of IFNα in the treatment of MPN patient-derived BM**

(**a**) Left: Dose-response matrix showing % inhibition (upper panel) and %CD41 expression (lower panel). Right: ZIP-Synergy score of inhibition (upper panel) and CD41 expression (lower panel) of *CALR^del52^* HPC^LSK^ upon treatment with 0-200nM palbociclib and 0-600ng pegIFNα. n=2 per condition.

(**b**) Short-term treatment of patient-derived BMMNCs of *JAK2^V617F^* (orange, patient #1 and #3), *CALR* Type 1 (red, patient #4, #5 and #6) and control (grey, patient #8 and #9) patients using 200nM palbociclib, 600ng pegIFNα, in combination or PBS control (left) and in lower dosages with 100nM palbociclib, 200ng pegIFNα, in combination or PBS control (right). Data represents calculated log2 fold-changes of HSPC (CD34^+^ CD38^-^) numbers with treatment conditions normalized to PBS controls within BMMNCs in liquid culture, using flow cytometry. Each line represents one patient sample, each dot a treatment condition within the sample. Statistical analysis was performed using a mixed-effects model as described in the methods. **P* <0.05; ***P* <0.01; ***P <0.001; ****P <0.0001.

(**c**) Representative picture of colony formation assays from patient-derived BMMNCs in methylcellulose treated with PBS control, 200nM palbociclib, 600ng peg-IFNα or in combination, respectively.

(**d**) Summary bar plot of short-term treatment of patient-derived BMMNCs of *JAK2^V617F^*, *CALR* Type 1 and control patients using 200nM palbociclib, 600ng pegIFNα, in combination or PBS control (upper panel) and in lower dosages with 100nM palbociclib, 200ng pegIFNα, in combination or PBS control (lower). Data represents % of living HSPCs (CD34^+^ CD38^-^) with treatment conditions normalized to PBS controls within BMMNCs in liquid culture, using flow cytometry. Each dot represents a patient sample in a condition.

(**e**) Flow cytometric analysis of colonies from patient-derived BMMNCs from *JAK2^V617F^*, *CALR* Type 1 and control patients using 200nM palbociclib, 600ng pegIFNα, in combination or PBS control. Upper panel: Percentages of HSPC (CD34^+^ CD38^-^) populations within colonies. Lower panel: Percentages of HPC (CD34^+^ CD38^+^) populations within colonies. Each dot represents a patient sample in a condition. The treatment prior to analysis was done for 14 days.

Error bars represent mean±SD. n≥2 per condition. **P* <0.05; ***P* <0.01 by two-way ANOVA followed by Tukey’s multiple comparison test.

(**f**) Mean fluorescence intensity (MFI) of intracellular CDK6 levels in MPN patients (*JAK2^V617F^*, *CALR* Type 1) and control in CD34^+^ CD38^-^ HSPCs of BMMNCs at basal levels measured via flow cytometry (left). Simple linear regression analysis of CDK6 levels in HSPCs (CD34^+^ CD38^-^) measured by intracellular flow cytometry staining correlating with HSPC apoptosis (log2 fold-change). Mean fluorescence intensity (MFI) of CDK6 in MPN patients (*JAK2^V617F^*, *CALR* Type 1) and control were calculated in HSPCs of BMMNCs at basal levels. MFIs were then correlated with the log2 fold-change of living HSPCs after the treatments with 200nM palbociclib combined with 600ng pegIFNα. Fold changes were plotted against MFIs, MPN patient data was separated from control patients. Simple linear regression was calculated, R-squared and p-values were plotted (right).

(**g**) Colony numbers derived from colony formation assay of BMMNCs from *JAK2^V617F^* (orange, patient #1, #3, #14, #15, #16), *CALR* Type 1 (red, patient #4, #5, #6, #7, #17, #18), *CALR* Type 2 (green, patient #19, #20) and control (grey, patient #8, #9, #11, #12, #13) patients using 100nM palbociclib, 200ng pegIFNα, in combination or PBS control. Data represent calculated log2 fold-change of colony numbers with treatment conditions normalized to PBS controls within BMMNCs in fully supplemented methylcellulose, obtained by microscopic counting. Each line represents one patient BM sample, each dot a treatment condition within the sample. Statistical analysis was performed using a mixed-effects model as described in the methods. **P* <0.05; ***P* <0.01; ***P <0.001; ****P <0.0001.

(**h**) Variant allele frequency (VAF) revealing allelic burden of MPN patients. Graph represents mutational burden after colony formation assay of BMMNCs from *JAK2^V617F^* (left; patient #14, #15, #16), *CALR* Type 1 (right; patient #17, #18) and *CALR* Type 2 (right; patient #19, #20) patients treated with 100nM palbociclib, 200ng pegIFNα, in combination or PBS control. After 14 days of treatments, colony dishes were collected. Upon DNA isolation, PCR and purification, products were library-prepped and sent for next generation sequencing (NGS) to determine mutational burden. Each line represents a patient sample, each dot a condition within a sample.

(**i**) Short-term treatment of patient-derived BMMNCs of *CALR* Type 2 (patient #19 and #20, Supplementary Table S3) patients using 100nM palbociclib, 200ng pegIFNα, in combination or PBS control. Data represents calculated log2 fold-changes of HSPC (CD34^+^ CD38^-^) numbers with treatment conditions normalized to PBS controls within BMMNCs in liquid culture, using flow cytometry. Each line represents one patient sample, each dot a treatment condition within the sample.

(**j**) Flow cytometric analysis of colonies from patient-derived BMMNCs of *CALR* Type 2 patients using 100nM palbociclib, 200ng pegIFNα, in combination or PBS control. Left: Percentages of HSPC (CD34^+^ CD38^-^) populations within colonies. Right: Percentages of HPC (CD34^+^ CD38^-^) populations within colonies. Each dot represents a patient sample in a condition. The treatment prior to analysis was done for 14 days.

(**k**) Left: Flow cytometry staining of CD34^+^ CD38^-^ HSPCs of *JAK2^V617F^* (orange), *CALR* Type 1 (red) and *CALR* Type 2 (green) MPN patient BMMNCs (Supplementary Table S3) after 3 days of liquid culture showing mean fluorescence intensity (MFI) of IFNAR1 (top) and IFNAR2 (bottom) upon 100nM palbociclib exposure versus control. Right: Mean fluorescence intensity (MFI) of intracellular CDK6 levels in MPN patient BMMNCs (*JAK2^V617F^*, *CALR* Type 1 and *CALR* Type 2) and control correlating with IFNAR1 (top) and IFNAR2 (bottom) surface expression at basal levels measured via flow cytometry.

**SUPPLEMENTARY REFERENCES**

1. Doma E, Mayer IM, Brandstoetter T, et al. A robust approach for the generation of functional hematopoietic progenitor cell  lines to model leukemic transformation. *Blood Adv*. 2021;5(1):39-53. doi:10.1182/bloodadvances.2020003022

2. Galvin A, Weglarz M, Folz-Donahue K, et al. Cell Cycle Analysis of Hematopoietic Stem and Progenitor Cells by Multicolor Flow  Cytometry. *Curr Protoc Cytom*. 2019;87(1):e50. doi:10.1002/cpcy.50

3. Zheng S, Wang W, Aldahdooh J, et al. SynergyFinder Plus: Toward Better Interpretation and Annotation of Drug  Combination Screening Datasets. *Genomics Proteomics Bioinformatics*. 2022;20(3):587-596. doi:10.1016/j.gpb.2022.01.004

4. Yadav B, Wennerberg K, Aittokallio T, Tang J. Searching for Drug Synergy in Complex Dose-Response Landscapes Using an  Interaction Potency Model. *Comput Struct Biotechnol J*. 2015;13:504-513. doi:10.1016/j.csbj.2015.09.001

5. Kollmann S, Grausenburger R, Klampfl T, et al. A STAT5B-CD9 axis determines self-renewal in hematopoietic and leukemic stem  cells. *Blood*. 2021;138(23):2347-2359. doi:10.1182/blood.2021010980

6. Robert K, Francesco P, S. BA, et al. A Gain-of-Function Mutation of JAK2 in Myeloproliferative Disorders. *New England Journal of Medicine*. 2025;352(17):1779-1790. doi:10.1056/NEJMoa051113

7. Hagemann-Jensen M, Ziegenhain C, Chen P, et al. Single-cell RNA counting at allele and isoform resolution using Smart-seq3. *Nat Biotechnol*. 2020;38(6):708-714. doi:10.1038/s41587-020-0497-0

8. Bolger AM, Lohse M, Usadel B. Trimmomatic: a flexible trimmer for Illumina sequence data. *Bioinformatics*. 2014;30(15):2114-2120. doi:10.1093/bioinformatics/btu170

9. Smith T, Heger A, Sudbery I. UMI-tools: modeling sequencing errors in Unique Molecular Identifiers to improve  quantification accuracy. *Genome Res*. 2017;27(3):491-499. doi:10.1101/gr.209601.116

10. Informatics B. FastQC. https://www.bioinformatics.babraham.ac.uk/projects/fastqc/

11. Frankish A, Diekhans M, Ferreira AM, et al. GENCODE reference annotation for the human and mouse genomes. *Nucleic Acids Res*. 2019;47(D1):D766-D773. doi:10.1093/nar/gky955

12. Dobin A, Davis CA, Schlesinger F, et al. STAR: ultrafast universal RNA-seq aligner. *Bioinformatics*. 2013;29(1):15-21. doi:10.1093/bioinformatics/bts635

13. Okonechnikov K, Conesa A, García-Alcalde F. Qualimap 2: advanced multi-sample quality control for high-throughput sequencing  data. *Bioinformatics*. 2016;32(2):292-294. doi:10.1093/bioinformatics/btv566

14. Liao Y, Smyth GK, Shi W. featureCounts: an efficient general purpose program for assigning sequence reads  to genomic features. *Bioinformatics*. 2014;30(7):923-930. doi:10.1093/bioinformatics/btt656

15. Team P. RStudio: Integrated Development Environment for R. *Posit Software, PBC*. Preprint posted online 2020. http://www.posit.co/

16. Team RC. R: A Language and Environment for Statistical Computing. *R Foundation for Statistical Computing*. Preprint posted online 2020. https://www.r-project.org/

17. Love MI, Huber W, Anders S. Moderated estimation of fold change and dispersion for RNA-seq data with DESeq2. *Genome Biol*. 2014;15(12):550. doi:10.1186/s13059-014-0550-8

18. Mootha VK, Lindgren CM, Eriksson KF, et al. PGC-1α-responsive genes involved in oxidative phosphorylation are coordinately downregulated in human diabetes. *Nat Genet*. 2003;34(3):267-273. doi:10.1038/ng1180

19. Subramanian A, Tamayo P, Mootha VK, et al. Gene set enrichment analysis: A knowledge-based approach for interpreting genome-wide expression profiles. *Proceedings of the National Academy of Sciences*. 2005;102(43):15545-15550. doi:10.1073/pnas.0506580102

20. Liberzon A, Birger C, Thorvaldsdóttir H, Ghandi M, Mesirov JP, Tamayo P. The Molecular Signatures Database (MSigDB) hallmark gene set collection. *Cell Syst*. 2015;1(6):417-425. doi:10.1016/j.cels.2015.12.004

21. Liberzon A, Subramanian A, Pinchback R, Thorvaldsdóttir H, Tamayo P, Mesirov JP. Molecular signatures database (MSigDB) 3.0. *Bioinformatics*. 2011;27(12):1739-1740. doi:10.1093/bioinformatics/btr260
